# Peptide Bond Formation Between the Hetrosubunits of ω-Transaminase, Alanine Dehydrogenase, and Formate Dehydrogenase Through Subunit Splicing Promoted by Heterodimerization of Leucine Zipper Motifs

**DOI:** 10.3389/fbioe.2020.00686

**Published:** 2020-06-30

**Authors:** Rong Li, Yao Chen, Kun Du, Wei Feng

**Affiliations:** Department of Biological Engineering, Beijing University of Chemical Technology, Beijing, China

**Keywords:** R-ω-transaminase, alanine dehydrogenase, formate dehydrogenase, hetrosubunit splicing, leucine zipper motifs

## Abstract

For the multimeric enzymes R-ω-transaminase (RTA), alanine dehydrogenase (AlaDH), and formate dehydrogenase (FDH), peptide bond formation between the hetrosubunits has been achieved by the intein-mediated *in vivo* subunit splicing. The subunit ligation is triggered by the heterodimerization of an arginine rich leucine zipper motif with a glutamic acid rich leucine zipper motif. The one-by-one ligation of hetrosubunits constructs the pairing enzymes RTA&AlaDH and AlaDH&FDH. The ligation modes were analyzed based on blue native polyacrylamide gel electrophoresis (BN-PAGE). The spectra of circular dichroism (CD), fluorescence, and two-dimensional FTIR provide information on the secondary structures and stability of the pairing enzymes. The enzyme-substrate interaction was analyzed based on microscale thermophoresis analysis. In contrast to the mixed three enzymes RTA + AlaDH + FDH, the ligated enzymes RTA&AlaDH + AlaDH&FDH exhibited a much larger substrate affinity, higher stability, and significantly enhanced activity.

## Introduction

Chiral amines are used for the synthesis of pharmaceuticals (Hannes et al., 2014; Simon et al., 2014). The optically pure amines have been used for the synthesis of biologically active compounds (Koszelewski et al., 2009). ω-Transaminases (ω-TAs) are multimeric enzymes and have been investigated for preparation of chiral amines (Han et al., 2019; Rocha et al., 2019; Isupov et al., 2019; Liao et al., 2019). ω-Transaminases are efficient biocatalysts for reductive amination of ketones to corresponding amines (Kelly et al., 2018; Padrosa et al., 2019). Alanine is commonly used as amine donor and the coproduct is pyruvate (Pressnitz et al., 2013). However, equilibrium is unfavored for the reductive amination. For circumventing this issue, ω-TAs have been combined with various enzymes to establish multienzyme networks for removing the coproduct, combining with lactate dehydrogenase (LDH) to convert pyruvate into lactic acid, incorporating pyruvate decarboxylase (PDC) to convert pyruvate to acetaldehyde and CO_2_ (Shin and Kim, 1999; Hohne et al., 2008). 2-Propanamine was also used as a cheap and achiral amine donor (Cassimjee et al., 2010). Yeast alcohol dehydrogenase was used for removing the coproduct acetone to displace the equilibrium.

**FIGURE 1:**
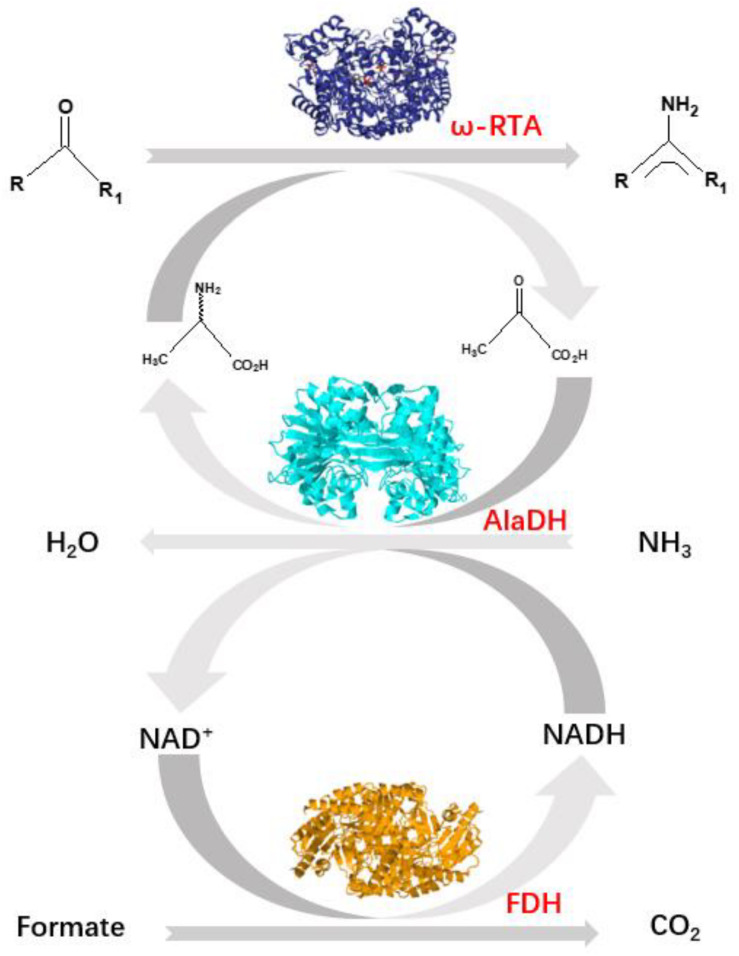
Shifting the equilibrium in RTA catalyzed reactions with alanine dehydrogenase (AlaDH) in combination with formate dehydrogenase (FDH).

Herein, we pay attention to another approach using an alanine dehydrogenase (AlaDH) to cooperate with *R*-ω-transaminase (RTA). In this approach, alanine is used as amine donor. The coproduct pyruvate is recycled back to alanine by consuming ammonia and formate (Koszelewski et al., 2008) (Figure 1). Formate dehydrogenase is integrated into the network to recycle NADH, which is needed for the reductive amination of pyruvate to alanine. However, we found that for some ketones, like acetophenone and 2-octanone, the conversions are very low under the catalysis of the mixed enzymes RTA + AlaDH + FDH. When these three enzymes are simply mixed together, the orientation and distance between the enzymes cannot be controlled. It is speculated that for efficient conversion of those ketones, the amine donor alanine and coproduct pyruvate as well as NADH and NAD^+^ must be efficiently transferred between RTA and AlaDH and between AlaDH and FDH, respectively. The distances between RTA and AlaDH and between AlaDH and FDH affect the transfer efficiency. Ideally, the enzymes are in close proximity with a molecular distance. Through gene fusion, two enzymes can be linked together (Lindbladh et al., 1994). However, for these multimeric enzymes, gene fusion does not work (exhibiting no activity). It is possibly because the fusion enzyme cannot fold correctly in the *Escherichia coli* cell.

Except the above mentioned low catalysis efficiency by the mixed enzymes, there is a significant concern for enzyme stability (Börner et al., 2017; Santos et al., 2018). The enzymes RTA, AlaDH, and FDH are multimeric enzymes. Experimental conditions may weaken the subunit-subunit interactions for multimeric enzymes (Fernandez, 2009). A sufficient operational stability of the enzymes is necessary for high productivity in chiral amine synthesis.

Previously, we have specifically ligated two multimeric enzymes with native peptides through intein-mediated *in vivo* subunit splicing (Du et al., 2017; Li et al., 2018). Herein, we develop the methodology, by using the coiled-coil association of an arginine-rich leucine zipper motif (Z_R_) (Moll et al., 2001) and a glutamic acid-rich leucine zipper motif (Z_E_) (Moll et al., 2001) to trigger the intein-mediated *in vivo* subunit splicing. RTA is specifically ligated to AlaDH and AlaDH is specifically linked to FDH with peptide bonds. The specific ligations were achieved through *in vivo* subunit splicing. Thus, the cycle of alanine → pyruvate → alanine between RTA and AlaDH and the cycle of NADH → NAD^+^→ NADH between AlaDH and FDH are formed. Evaluation of the three-enzyme system has been performed in terms of enzyme secondary structure, enzyme stability, and catalysis efficiency.

## Materials and Methods

### Materials

DNA ligase, DNA polymerase, and restriction enzymes were purchased from Thermo Fisher Scientific. Oligonucleotide primers were synthesized by BioMed Tech. *Escherichia coli* strain DH5α used as a host for DNA manipulation and strain BL21 (DE3) as a host for expressing enzymes were both purchased from Zoman Bio. And all other reagents were purchased from Sigma-Aldrich (Shanghai, China).

### Construction of Plasmids

The alanine dehydrogenase (AlaDH) gene was synthesized based on the genome sequence of *Archaeoglobus fulgidus* (EC:1.4.1.1) (Agren et al., 2008) and cloned in pUC57 plasmid by Genewiz (Suzhou, China). The AlaDH gene was purified using a DNA extraction kit (Omega Bio-tek). The purified AlaDH gene was digested with both *BamHI* and *EcoRI* restriction endonucleases, which then was ligated into the plasmid pETDuet-1 cleaved with *BamHI* and *EcoRI* restriction endonucleases already. The AlaDH gene was amplified by PCR using the paired primers AlaDH-fp and AlaDH-rp (Supplementary Table S1), at the amplification conditions (Supplementary Table S1). The Z_E_-Int^C^-link gene was amplified by PCR using the Z_E_-Int^C^-fp and Z_E_-Int^C^-rp primers (Supplementary Table S1). The purified Z_E_-Int^C^-link gene was digested with *NcoI* and *BamHI* restriction endonucleases, then was ligated into the vector AlaDH-pETDuet that had been cleaved with *NcoI* and *BamHI* restriction endonucleases. The constructed expression plasmid was designated as Z_E_-Int^C^-link-AlaDH-pETDuet.

The *R*-ω-transaminase (RTA) gene used here was the same as that in our previous work (Sun et al., 2018). The RTA gene was amplified by PCR using primers RTA-fp and RTA-rp (Supplementary Table S1). The purified RTA gene was digested with both *NdeI* and *KpnI* restriction endonucleases, and then was ligated into the plasmid Z_E_-Int^C^-link-AlaDH-pETDuet, which had been cleaved with *NdeI* and *KpnI* restriction endonucleases. Therefore, the plasmid Int^C^-link-AlaDH/RTA-pETDuet in *Escherichia coli* was constructed. The gene of link-Int^N^-Z_R_ was amplified by PCR using the Int^N^-Z_R_-fp and Int^N^-Z_R_-rp primers (Supplementary Table S1). The purified link-Int^N^-Z_R_ gene was digested with both *KpnI* and *XhoI* restriction endonucleases, and then was ligated into the plasmid Z_E_-Int^C^-link-AlaDH-pETDuet, which had been cleaved with *KpnI* and *XhoI* restriction endonucleases. Then plasmid for coexpressing the precursors RTA-link-Int^N^-Z_R_ and Z_E_-Int^C^-link-AlaDH was finally constructed. It is Z_E_-Int^C^-link-AlaDH/RTA-link-Int^N^-Z_R_-pETDuet, as illustrated in Supplementary Figure S1a. The FDH gene (Fogal et al., 2015) was amplified. The plasmid for coexpressing the precursors Z_E_-Int^C^-link-AlaDH and FDH-link-Int^N^-Z_R_ was constructed similarly, designated as Z_E_-Int^C^-link-AlaDH/FDH-link-Int^N^-Z_R_-pETDuet as illustrated in Supplementary Figure S1b.

For the hetrosubunit splicing without using the coiled-coil association of Z_E_ and Z_R_ to promote the splicing, the plasmids were constructed using the same procedures as described above. The plasmid for coexpressing the precursors RTA-link-Int^N^ and Int^C^-link-AlaDH was constructed, designated as Z_E_-Int^C^-link-AlaDH/RTA-link-Int^N^- Z_R_-pETDuet, as illustrated in Supplementary Figure S1c. The plasmid for coexpressing the precursors Int^C^-link-AlaDH and FDH-link-Int^N^ was constructed, designated as Int^C^-link-AlaDH/FDH-link-Int^N^-pETDuet as illustrated in Supplementary Figure S1d.

### Enzyme Expression and *in vivo* Subunit Splicing

The genes were transferred into *Escherichia coli* BL21 (DE3) cells. The cells were cultured in Luria-Bertani medium (50 μg/ml ampicillin) under shaking (220 rpm) at 37°C for 3 h. At this time, the OD_600_ reached about 0.4, and the cells grew at exponential phase. Then the cells were induced with 0.5 mM isopropyl β-D-1-thiogalactopyranoside (IPTG), and the culture temperature was changed to 25°C. At this temperature the cells grew for 8, 12, and 16 h, respectively. During the co-expression of the precursors Z_E_-Int^C^-link-RTA and AlaDH-link-Int^N^-Z_R_, the *in vivo* subunit splicing occurred simultaneously. Both SDS-PAGE and blue native polyacrylamide gel electrophoresis (BN-PAGE) were used to monitor and analyze the crude proteins released from the cells. It was found that 8 h is enough for the cell growth to accomplish the *in vivo* subunit splicing, determined by observing the bands for the splicing products.

The *E. coli* cells were collected after 30 min centrifugation at 7000 *g* at 4°C. The cells were then disrupted under ultrasonication on ice. The cell debris was removed by centrifugation at 7,000 *g* at 4°C for 30 min. The supernatant was collected into a fresh tube. The enzymes were first purified by passing through a chromatography column (GE Life Sciences) filled with Ni Sepharose beads (GE Life Sciences). Five hundred mM imidazole was used as the elution liquid, which was separated from the purified enzymes by dialysis into a buffer (500 mM NaCl, 50 mM Tris-Cl pH 7.5, 1 mM EDTA). The purified enzymes were further fractionated by ultrafiltration (GE Healthcare, 50000 Da cut off). The splicing products were finally purified using a gel filtration chromatography (AKTA Pure chromatography system, GE Healthcare, Superose 6 column).

### Microscale Thermophoresis (MST)

The experiments were performed on an instrument of microscale thermophoresis (MST) (Nano Temper technologies). The splicing product RTA&AlaDH was not labeled by fluorescence. The dialysis liquid consisted of 50 mM Tris-Cl (PH 8.0), 150 mM NaCl, and 10 mM MgCl_2_. After dialysis, RTA&AlaDH (200 nM) was mixed with the ligand 2-octanone (100 mM), and then incubated at 30°C for an hour. A 16 step dilution series from 10 mM to 0.25 μM were prepared by DMSO. 10 μL of each step was filled into 16 fresh small micro reaction tubes and was well mixed by pipetting up and down. The measurements were performed at 20% IR-laser power and 40% MST power. Using the software MO. Affinity Analysis, the binding coefficient Kd values were obtained by fitting the fraction of bound protein to the quadratic solution of the binding reaction equilibrium. MST benefits from very low sample consumption and short measurement times.

### Attenuated Total Reflectance Fourier Transform Infrared (ATR-FTIR)

FTIR- attenuated total reflection (ATR) spectra were recorded on a spectrometer (Bruker Tensor 27) at a nominal resolution of 2 cm^–1^. Each spectrum is the result of the accumulation and averaging of 512 interferograms. Ultrapure nitrogen was continuously introduced into the sample compartment to minimize the spectral contribution of atmospheric water. The enzyme was dissolved in 10 mM deuterated potassium phosphate buffer (pH 7.4) with a concentration of 15 mg/mL. The enzyme solution was rapidly prepared at 10°C, thus condensation of atmospheric water into the solution can be avoided. D_2_O was added into the sample of the enzyme solution, and the measurement of FTIR spectra was started. The hydrogen-deuterium exchange experiment was carried out for 90 min. The pure spectrum of enzyme was obtained by subtracting the spectra of D_2_O.

### Enzymatic Activity

For transformation of the amines, the enzymatic catalysis was performed at 30°C under shaking at 200 rpm. The enzyme concentrations were: 0.5 mg/mL RTA&AlaDH + 0.5 mg/mL AlaDH&FDH; 0.5 mg/mL RTA#AlaDH + 0.5 mg/mL AlaDH#FDH. The concentration of substrate 2-octanone was 50 mM. The substrate solution was prepared by dissolving in phosphate buffered solution (mix 100 mM Na_2_HPO_4_ and 100 mM NaH_2_PO_4_ in proportion to pH 8.0), then alanine (5 mM), pyridoxal-5′-phosphate (1 mM), 1 mM NAD^+^, and 100 mM ammonium formate were added. The reaction volume was 2 mL. The chromatographic pure methanol and ultra pure water were respectively filtered by the filter membrane, and then the ultrasonic treatment was carried out to exhaust bubbles for half an hour. The concentration of the substrate 2-octanone was monitored by HPLC (Shimadzu LC-10A) with ultraviolet detector at 280 nm using a C18 column (Diamonsil C18 250 × 4.6 mm, 5 μm). Setup column thermostat at 30°C, and the mobile phase was methanol/water (60:40 by vol) at 0.8 mL/min, and the injected sample was 20 μL.

## Results and Discussion

### *In vivo* Hetrosubunit Splicing

**FIGURE 2:**
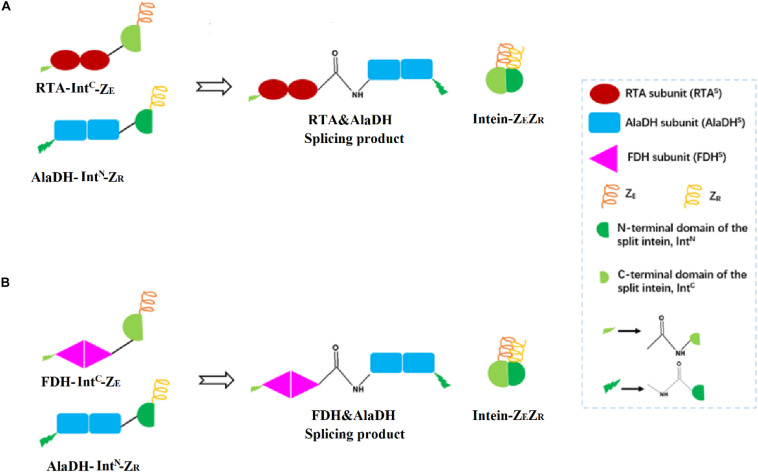
Schematic presentation for the ligations of multienzymes with peptide bonds through hetrosubunit splicing triggered by the dimerization of ZE and ZR. **(A)** The RTA subunit (RTA^S^) is ligated to the AlaDH subunit (AlaDH^S^) forming the splicing product RTA&AlaDH. **(B)** The FDH subunit (FDH^S^) is ligated to AlaDH^S^ forming the splicing product FDH&AlaDH. ZR, arginine-rich leucine zipper; ZE, glutamic acid-rich leucine zipper.

Herein, the split intein was utilized to specifically ligate multimeric enzymes for potential industrial application. These multimeric enzymes have large molecular weights. The ligations were accomplished through intein-mediated *in vivo* subunit splicing, triggered by the coiled-coil association of an arginine-rich leucine zipper motif (Z_R_) and a glutamic acid-rich leucine zipper motif (Z_E_) (Moll et al., 2001). The developed methodology was used to ligate the enzymes for constructing three-enzyme network, as illustrated in Figure 2.

*R*-ω-Transaminase (RTA) and alanine dehydrogenase (AlaDH) are dimeric enzymes, each consisting of two subunits. ZE was fused to the C-terminal domain of the split NpuDnaE intein (Int^C^), the ZE-Int^C^ segment was fused to the N-terminus of the RTA subunit (RTA^S^), constructing ZE-Int^C^-RTA^S^. ZR was fused to the N-terminal domain of the split NpuDnaE intein (Int^N^), the ZR-Int^N^ segment was fused to the C-terminus of the AlaDH subunit (AlaDH^S^), constructing ZR-Int^N^-AlaDH^S^ (Shah et al., 2011). In between ZR-Int^N^ and AlaDH^S^ and in between ZE-Int^C^ and RTA^S^, rigid linkers (EPPPPLPPPPLPPPPEPPPP) are required, in order to reduce the steric hindrance for the subunit splicing. Dimerization of ZE and ZR can naturally occur due to the coiled-coil association between ZE and ZR (Shah et al., 2011). Herein, the dimerization of ZE and ZR was utilized to promote the self-excision of Int^C^ and Int^N^ and concomitant ligation of the subunits of RTA^S^ and AlaDH^S^. The splicing product is designated as RTA&AlaDH (Figure 2A). Another splicing product FDH&AlaDH is obtained similarly, as shown in Figure 2B. Thus, the cycle of alanine → pyruvate → alanine between RTA and AlaDH and the cycle of NADH → NAD^+^→ NADH between AlaDH and FDH are formed (Figure 1).

**FIGURE 3:**
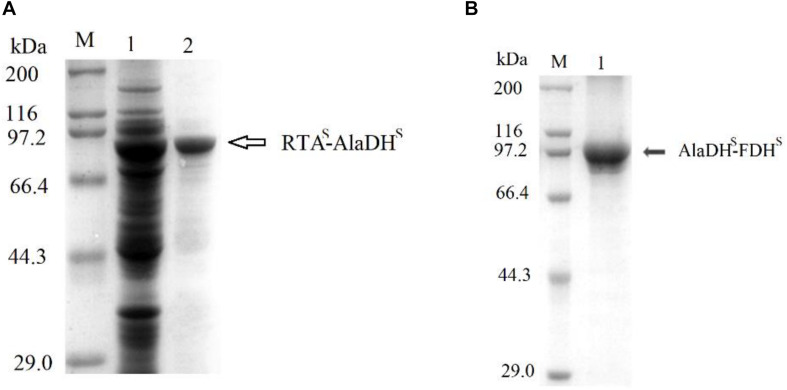
SDS-PAGEs for the splicing products. **(A)** Lane 1 for total proteins, lane 2 for RTA&AlaDH. **(B)** Lane 1 for AlaDH&FDH. M, marker protein.

The splicing products RTA&AlaDH and AlaDH&FDH were purified sequentially by Ni-Sepharose beads, ultrafiltration fractionation, and gel filtration chromatography. They were analyzed through SDS-PAGE (Figure 3). The band indicated by the black arrow in Figure 3A is ascribed to the ligation of the RTA subunit (RTA^S^) with the AlaDH subunit (AlaDH^S^), the ligated subunit of RTA^S^-AlaDH^S^. The molecular weight of RTA^S^-AlaDH^S^ is 75.9 kDa. The band of RTA^S^-AlaDH^S^ is below the position 97.2 kDa of a mark protein. The band indicated by the black arrow in Figure 3B is attributed to the ligation of AlaDH^S^ with the FDH subunit (FDH^S^). The band of the ligated subunit AlaDH^S^-FDH^S^ has a molecular weight of 83.1 kD. Appearance of the bands of RTA^S^-AlaDH^S^ and AlaDH^S^-FDH^S^ confirmed the ligations of RTA^S^ with AlaDH^S^ and AlaDH^S^ with FDH^S^, through the *in vivo* posttranslational hetrosubunit splicing triggered by the dimerization of ZE and ZR.

### Ligation Mode Based on BN-PAGE Analysis

**FIGURE 4:**
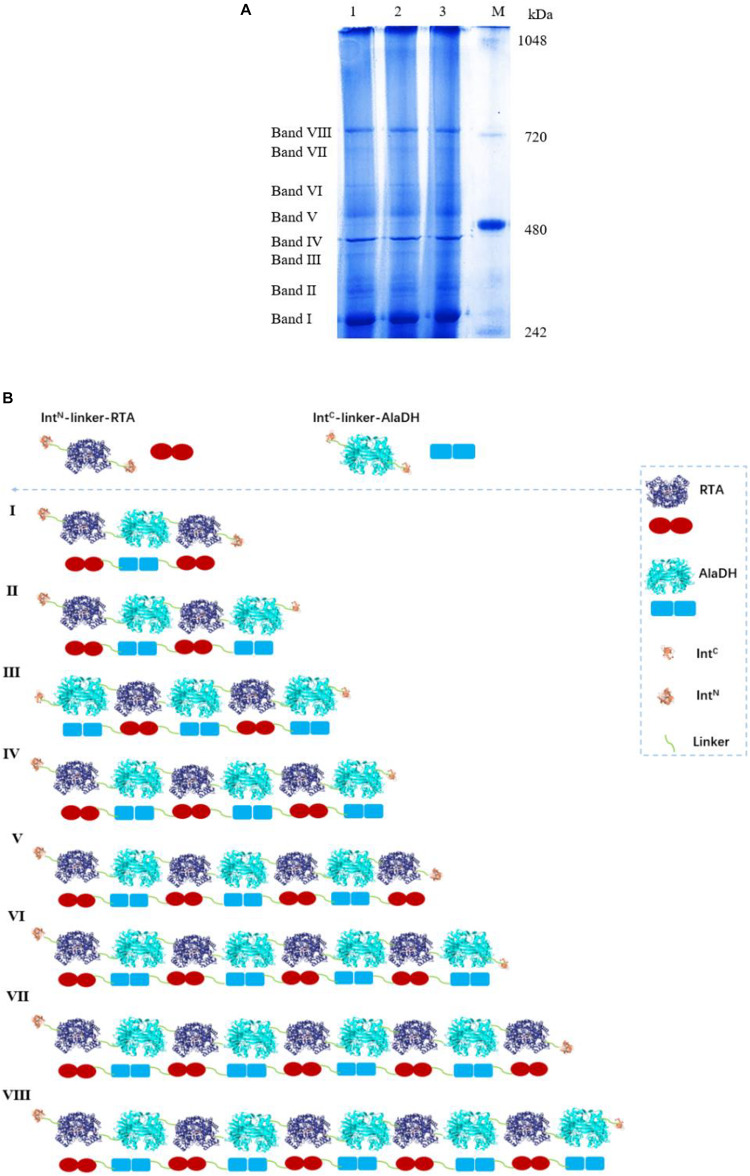
**(A)** Analysis of the splicing product RTA&AlaDH by BN-PAGE showing eight bands. Lane M: marker. Lanes 1, 2, 3 are for three samples. **(B)** Schematic diagram representing the RTA&AlaDH fractions derived from the ligation modes.

We have tried to use matrix-assisted laser desorption and ionization time-of-flight mass spectrometry (MALDI-TOF-MS) to identify the fractions of the splicing products RTA&AlaDH and AlaDH&FDH. However, the measured data are not useful, due to the lack of reliability of the data. This is ascribed to the large molecular weights of the fractions of RTA&AlaDH and AlaDH&FDH. In an alternative approach, blue native polyacrylamide gel electrophoresis (BN-PAGE) was used to analyze RTA&AlaDH and AlaDH&FDH. BN-PAGE has been proved to be effective for identifying proteins from multiprotein complexes (Wiedemann et al., 2003; Wittig et al., 2016). The BN-PAGE of RTA&AlaDH is shown in Figure 4A. Eight bands appeared. It is meant that RTA&AlaDH comprises eight fractions. Marker proteins are shown on Lane M. Based on the bands on lane M, the electrophoretic mobility of the marker proteins is plotted against the molecular weight (Supplementary Figure S2), and an equation was obtained. The apparent molecular weights (AMWs) corresponding to the eight bands were estimated, based on the comparison between the electrophoretic mobility of marker proteins and that of the eight bands. The AMWs of the bands I, II, III, IV, V, VI, VII, and VIII were determined to be 254.8, 311.9, 388.0, 422.4, 480.7, 564.9, 674.2, and 741.3 kDa, respectively (Supplementary Table S2). Based on the band intensity, the relative abundances of the eight bands were determined to be 26.5, 2.3, 5.8, 20.4, 11.8, 6.8, 7.6, 19.1% for bands I, II, III, IV, V, VI, VII, and VIII, respectively. For each fraction of RTA&AlaDH, the mode of hetrosubunit ligation was searched. The criterion is that the theoretical molecular weight (TMW) predicated on the basis of the ligation mode is in accordance with the AMW. Based on the molecular weights of intein, ZR-Int^N^-RTA^S^, and ZE-Int^C^-AlaDH^S^, the TMW of the possible ligation mode were predicated. Supplementary Table S2 lists those ligation modes with TMWs being in accordance with the AMWs of the eight bands. The ligation modes were finally determined to be, RTA∼AlaDH∼RTA (band I), RTA∼AlaDH∼RTA∼AlaDH (band II), AlaDH∼RTA∼ AlaDH∼RTA∼AlaDH (band III), RTA∼AlaDH∼RTA∼AlaDH∼RTA∼AlaDH (band IV), RTA∼ AlaDH∼RTA∼AlaDH∼RTA∼AlaDH∼RTA (band V), AlaDH∼ RTA∼ AlaDH∼RTA∼AlaDH∼RTA∼AlaDH (band VI), RTA∼ AlaDH∼RTA∼AlaDH∼RTA∼ AlaDH∼RTA∼AlaDH (band VII), RTA∼AlaDH∼RTA∼AlaDH∼RTA∼AlaDH∼RTA ∼Ala DH∼RTA∼AlaDH (band VIII).

Figure 4B schematically illustrates the ligation modes corresponding to the eight bands. The paired subunit RTA^S^-AlaDH^S^ (as illustrated by in Figure 4B) is formed by the hetrosubunit ligation with a peptide bond through the subunit splicing, and it is the key subunit in the RTA&AlaDH fractions. Based on the relative abundances and eight ligation modes, the amount of paired subunit RTA^S^-AlaDH^S^ is determined to be 5.4 mol per mole of RTA&AlaDH.

For comparison, the subunit splicing was carried out without using the promotion by the heterodimerization of ZR and ZE. The obtained SDS-PAGE (Supplementary Figure S3a) is similar to that in Figure 3, confirming the ligation of RTA with AlaDH through the *in vivo* subunit splicing. The splicing product is designated as RTA#AlaDH. The BN-PAGE analysis is shown in Supplementary Figure S3b. Four bands appeared, with apparent molecular weights being 271.2, 346.7, 435.1, and 761.7 kDa, corresponding to bands I, II, III, and IV, respectively, and the relative abundances of the four bands are 25.4, 43.9, 10.1, 20.6%, respectively. The ligation modes are shown in Supplementary Figure S3c. The amount of paired subunit RTA^S^-AlaDH^S^ is determined to be 4.2 mol per mole of RTA#AlaDH. The amount of paired subunit RTA^S^-AlaDH^S^ in RTA&AlaDH is 28.5% higher than that in RTA#AlaDH.

**FIGURE 5:**
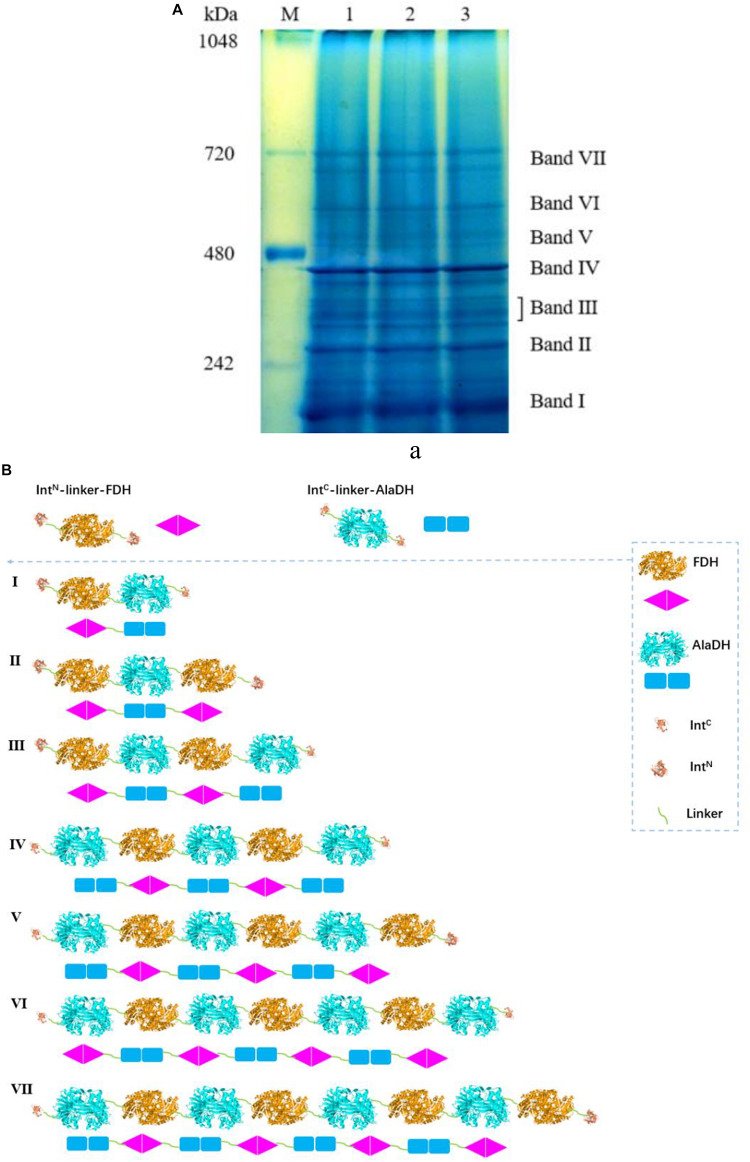
**(A)** Analysis of the splicing product AlaDH&FDH by BN-PAGE showing seven bands. Lane M: marker. Lanes 1, 2, 3 are for three samples. **(B)** Schematic diagram representing the AlaDH&FDH fractions derived from the ligation modes.

The BN-PAGE for AlaDH&FDH shown in Figure 5A. The seven bands correspond to seven fractions. Based on Supplementary Figure S4, the AMWs of the bands I, II, III, IV, V, VI, and VII were determined to be 210.6, 278.7, 327.0, 399.4, 469.7, 533.8, and 665.1 kDa, respectively. The relative abundances of the seven bands are 27.4, 20.4, 14.8, 24.3, 3.4, 3.8, and 5.9%, respectively. The ligation modes were, FDH∼AlaDH (band I), FDH∼AlaDH∼FDH (band II), FDH∼AlaDH∼FDH∼AlaDH (band III), AlaDH∼FDH∼AlaDH∼FDH∼AlaDH (band IV), AlaDH∼FDH∼AlaDH∼FDH∼AlaDH∼FDH (band V), AlaDH∼FDH∼AlaDH∼ FDH∼AlaDH∼FDH∼AlaDH (band VI), AlaDH∼FDH∼AlaDH∼FDH∼AlaDH∼FDH∼ AlaDH∼FDH (band VII). Figure 5B schematically illustrates the ligation modes corresponding to the seven bands. The paired subunit AlaDH^S^-FDH^S^ is indicated by in Figure 5B. Based on the relative abundances and seven ligation modes, the amount of paired subunit AlaDH^S^-FDH^S^ is determined to be 2.9 mol per mole of AlaDH&FDH. The splicing product AlaDH#FDH was obtained without using the promotion by the heterodimerization of ZR and ZE. The SDS-PAGE (Supplementary Figure S5a) confirmed the ligation of AlaDH with FDH through the *in vivo* subunit splicing. The BN-PAGE for AlaDH#FDH is shown in Supplementary Figure S5b. Five bands appeared, with apparent molecular weights being 205.8, 280.9, 331.6, 403.8, and 679.3 kDa, corresponding to bands I, II, III, IV, and V, respectively, and the relative abundances of the five bands are 24.4, 22.7, 20.7, 27.4, and 4.8%, respectively. The ligation modes are shown in Supplementary Figure S5c. The amount of paired subunit AlaDH^S^-FDH^S^ is 2.8 mol per mole of AlaDH#FDH. The amount of paired subunit RTA^S^-AlaDH^S^ in AlaDH&FDH is comparable to that in AlaDH#FDH.

### Alpha-Helical Content Analysis by Circular Dichroism (CD) Spectra

**FIGURE 6:**
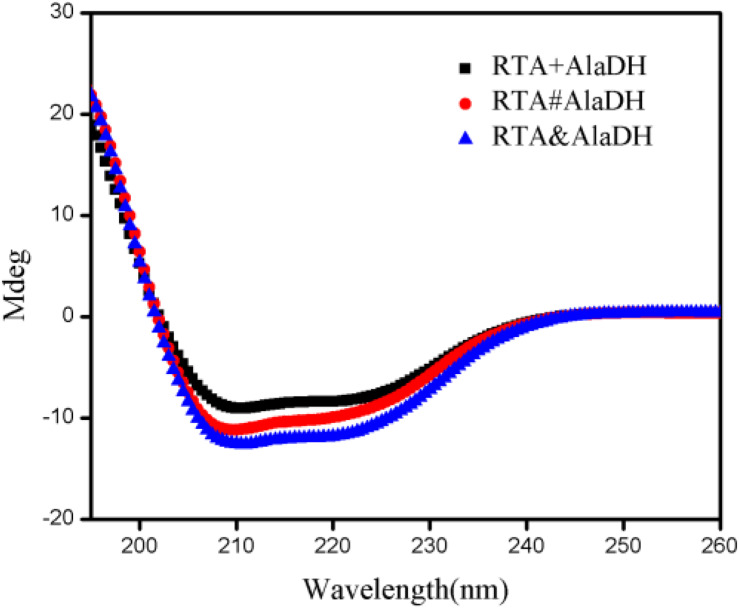
CD spectra for the splicing products RTA&AlaDH and RTA#AlaDH and the mixed enzymes RTA + AlaDH.

Here we presented two splicing products. RTA&AlaDH was obtained through the *in vivo* hetrosubunit splicing triggered by the coiled-coil association of Z_E_ and Z_R_. For comparison, RTA#AlaDH was obtained through the *in vivo* hetrosubunit splicing without using the coiled-coil association of Z_E_ and Z_R_. CD spectra were measured for the splicing products RTA&AlaDH and RTA#AlaDH (Figure 6), with the CD spectrum of the mixed enzymes RTA + AlaDH as control. The CD absorption at 222 nm is sensitive to the change of alpha-helical content of proteins (Fasshauer et al., 1997). In comparison to RTA + AlaDH, both RTA&AlaDH and RTA#AlaDH exhibited a more negative ellipticity at 222 nm (Figure 6). The CD spectra demonstrated that the splicing products RTA&AlaDH and RTA#AlaDH contain more alpha-helical structure than the mixed enzymes RTA+AlaDH. This is possibly ascribed to the formation of paired subunit RTA^S^-AlaDH^S^ in the splicing products. The CD spectra also show that RTA&AlaDH contains more alpha-helical structure than RTA#AlaDH. Supplementary Figure S6 shows the CD spectra for the splicing products AlaDH&FDH and AlaDH#FDH and the mixed enzymes AlaDH + FDH. The difference between the spectra is not significant.

### Analysis of Enzyme Structures by Two-Dimensional FTIR Spectra

The CD spectra (Figure 6) showed that the splicing products RTA&AlaDH and RTA#AlaDH comprise more alpha-helical structure then the mixed enzymes RTA+AlaDH. Herein, to further get insight into the structures of the splicing products, RTA&AlaDH and RTA#AlaDH as well as RTA + AlaDH were subjected to the measurement of two-dimensional FTIR spectra, as described in the experimental section. Through two-dimensional correlation spectroscopy, individual bands with a high resolution can be identified. Upon external perturbation, the sequential order of spectral intensity changes of the enzyme structures (unordered, α-helix, and β-sheet) can be obtained. By combining the sign of the peaks in the synchronous and asynchronous maps, the sequence of the spectral variations can be determined (Noda, 1989).

**FIGURE 7:**
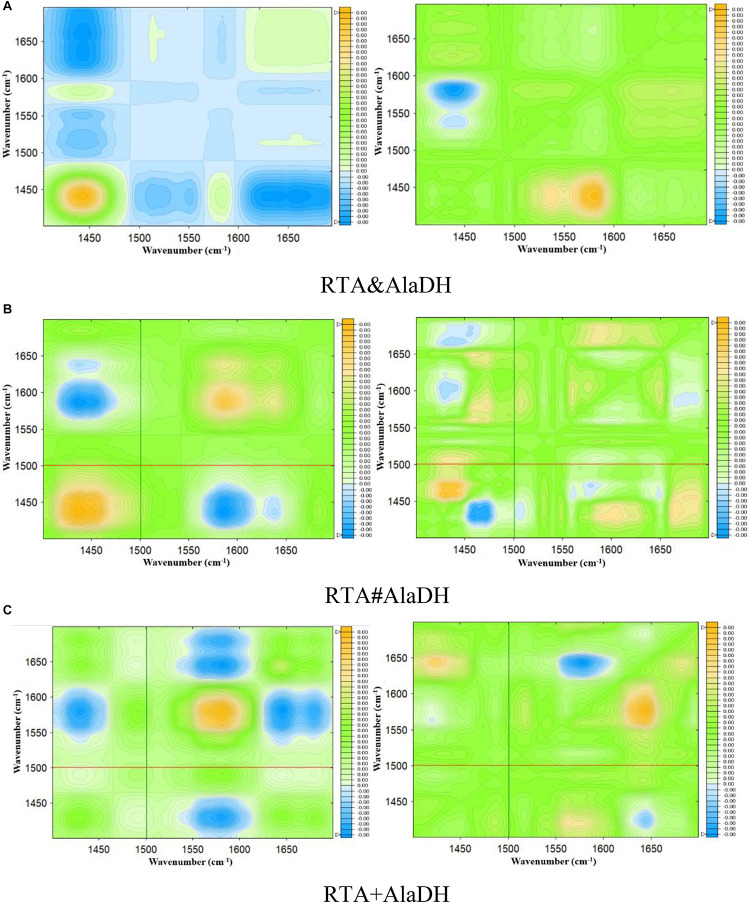
Synchronous (left) and asynchronous (right) 2D correlation spectra of the enzymes obtained for 90 min of deuteration. **(A)** Splicing product RTA&AlaDH; **(B)** splicing product RTA#AlaDH; **(C)** mixed enzyme RTA + AlaDH.

Infrared spectra as a function of the deuteration time were measured as shown in Supplementary Figure S7. Based on Supplementary Figure S7, two-dimensional spectra are obtained for RTA&AlaDH (Figure 7A), RTA#AlaDH (Figure 7B), and RTA + AlaDH (Figure 7C). During the measurements, D_2_O was added, hence hydrogen-deuterium (H-D) exchange was the external perturbation. The main correlation peaks observed in the synchronous and asynchronous maps of Figure 7 are summarized in Supplementary Table S3. Published literature on infrared spectroscopy of proteins provides the basis for the band assignment (Richard et al., 2005; Mecozzi et al., 2011). The sequential order of H-D exchange is determined based on Noda’s rules (Noda, 1989). For RTA&AlaDH (Figure 7A), the cross-peaks (1650, 1450) and (1610, 1450) can be identified from the synchronous and asynchronous maps. The Noda’s rules confirm that the unordered structure (1450 cm^–1^) exhibited a faster H-D exchange than the α-helix (1650 cm^–1^) and β-sheet (1610 cm^–1^) structures. For RTA#AlaDH, the cross-peaks (1639, 1430) and (1665, 1437) can be identified from the synchronous and asynchronous maps are shown Figure 7B. The unordered structure (1430 cm^–1^) exhibited a faster H-D exchange than the α-helix (1639 cm^–1^), and the unordered structure represented by 1437 cm^–1^ exchanged before the β-sheet structure (1665 cm^–1^). For RTA + AlaDH (Figure 7C), the cross-peaks (1645, 1425) and (1680, 1427) can be identified. The unordered structure (1425 cm^–1^) exhibited a faster H-D exchange than the α-helix (1645 cm^–1^), and the unordered structure represented by 1427 cm^–1^ exchanged before the β-sheet structure (1680 cm^–1^).

The analysis of the two-dimensional FTIR spectra (Figure 7) demonstrated that the three two-enzyme systems, RTA&AlaDH, RTA#AlaDH, and RTA + AlaDH, exhibited the same sequence of the spectral variations upon the external perturbation of D_2_O. That is the unordered structures exhibited a faster H-D exchange than the structures of α- helices and β-sheets. Ascribing to the presence of strong hydrogen bonds in α-helices and β-sheets, the protons of α-helix and β-sheet are more difficult to exchange than those of unordered structures (Richard et al., 2005; Mecozzi et al., 2011). There is no regular hydrogen bonds in the unordered structures, the amide protons can be exchanged more easily than those of α-helices and β-sheets. The sequential order of H-D exchange demonstrated that the *in vivo* hetrosubunit splicing had little effect on the hydrogen bonding interactions presented, that maintain the secondary structures of α-helices and β-sheets of the enzymes. These secondary structures play an important role in the function of the enzymes.

### Anti-denaturation Through Hetrosubunit Splicing

**FIGURE 8:**
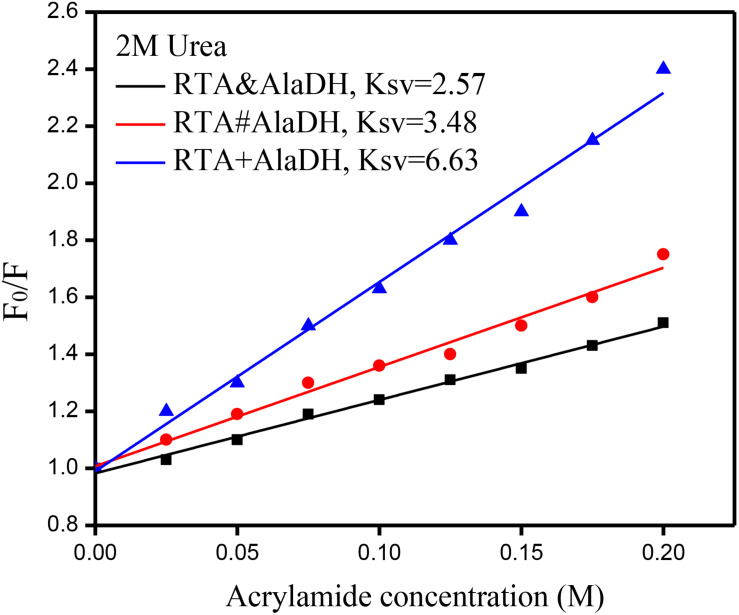
Stern–Volmer plots for the quenching of protein fluorescence by acrylamide. F_0_ and F are the fluorescence intensities in the absence and presence of acrylamide, respectively.

Multienzymes comprise of subunits that are associated through non-covalent interactions, which play an important role in maintaining the quaternary structures. However, the non-covalent interactions can be weakened or partially destroyed under certain experimental conditions. The dissociation of the subunits leads to the inactivation of multimeric enzymes. Multimeric enzymes face the concern of stability, especially when applied at industrial scale (Fernandez, 2009). For the two-enzyme systems, RTA&AlaDH, RTA#AlaDH, and RTA+AlaDH, fluorescence spectra were measured to investigate enzyme stability. Urea (2 M) was used as the denaturing agent. Acrylamide was used to quench the intrinsic fluorescence of the enzymes. The spectra of fluorescence intensity changing with the acrylamide concentration are shown in Supplementary Figure S8. Stern–Volmer equation F_0_/F = 1+ *K*_SV_[A] was used to evaluate the concentration-dependence quenching (Georlette et al., 2004). In the equation, F and F_0_ are the fluorescence intensities in the presence and absence of acrylamide, respectively. [A] is the acrylamide concentration. The quenching constant *K*_SV_ reflects the conformational change of enzymes (Georlette et al., 2004). The ratio of F_0_/F is plotted against acrylamide concentration as shown in Figure 8. A larger *K*_SV_ corresponds to a larger conformational change of the protein. The *K*_SV_ value of RTA&AlaDH is 2.57, which is smaller than 3.48 for RTA#AlaDH and much smaller than 6.63 for RTA + AlaDH. In comparison to RTA&AlaDH and RTA#AlaDH, RTA + AlaDH is more inclined to expose the tryptophan residues upon interfering by urea. The splicing products are stable than the mixed two enzymes RTA+AlaDH. The splicing products resulted from the covalent ligation of the RTA subunit with the AlaDH subunit, making the splicing products become more rigid than mixed enzymes. It is indicated that RTA&AlaDH is stable than RTA#AlaDH. Based on the BN-PAGE analysis (Figure 4 and Supplementary Figure S3b), the amount of the paired subunit RTA^S^-AlaDH^S^ in RTA&AlaDH is 28.5% higher than that in RTA#AlaDH. This results in a lower subunit mobility in RTA&AlaDH, leading to a more rigid structure. The stabilities of AlaDH&FDH, AlaDH#FDH, and AlaDH+FDH have also been analyzed based on the fluorescence intensity (in the legend of Supplementary Figure S9).

### Enzyme-Substrate Interaction Based on Microscale Thermophoresis Analysis

**FIGURE 9:**
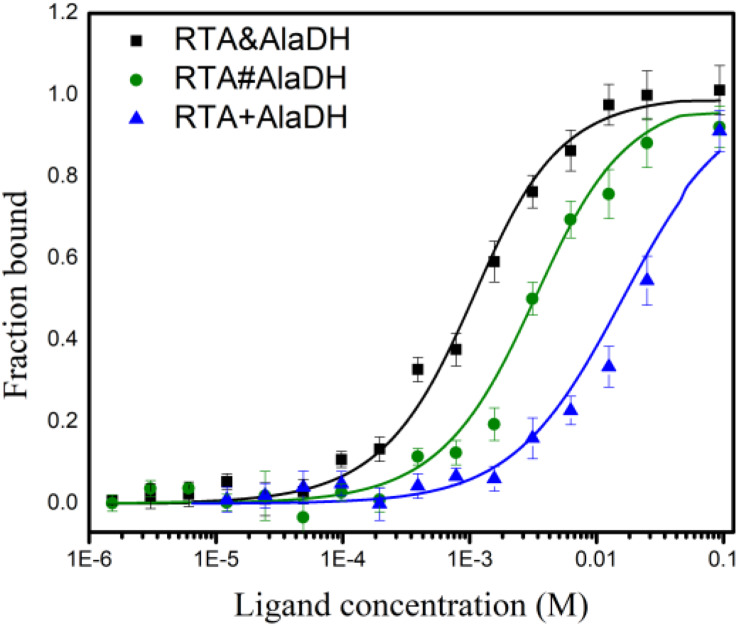
Binding of the two-enzyme systems to the substrate 2-octanone measured by MST. The curves are obtained from the specific change in the thermophoretic mobility upon 2-octanone (ligand) titration to the enzymes. The error bars represent the standard deviations of triplicate samples.

The analysis of enzyme binding to substrate can provide the information of enzyme affinity toward the substrate. Microscale thermophoresis (MST) is a relatively recent technique based on the unique physical principle of thermophoresis. It can be used to quantify the interactions of proteins and small molecules (Seidel et al., 2013). The two-enzyme systems, RTA&AlaDH, RTA#AlaDH, and RTA+AlaDH, were subjected to MST measurements to quantify binding coefficients (*Kd*). Figure 9 shows the fraction bound versus ligand concentration. The substrate 2-octanone was the ligand. RTA&AlaDH exhibited a *Kd* value of 0.68 ± 0.15 mM, which is smaller than the *Kd* value of 2.33 ± 3.22 mM by RTA#AlaDH, and much smaller than the *Kd* value of 14.91 ± 10.13 mM by RTA+AlaDH. It is indicated that RTA&AlaDH exhibited the affinity to 2-octanone 3.4 times stronger than that by RTA#AlaDH, and 6.5 times stronger than that by RTA+AlaDH.

### Enzymatic Activity

**FIGURE 10:**
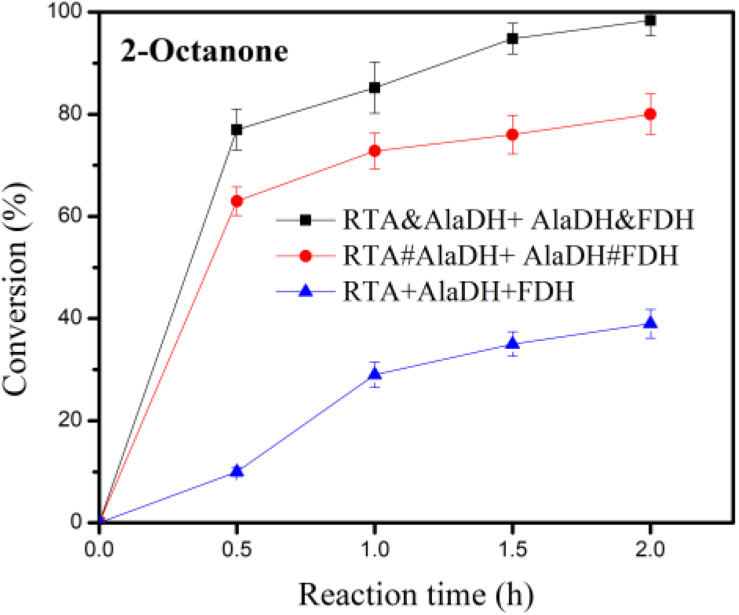
Conversion of 2-octanone under the catalysis of RTA&AlaDH + AlaDH&FDH, RTA#AlaDH + AlaDH#FDH, and RTA + AlaDH + FDH. The error bars represent the standard deviations of triplicate samples.

In the conversion of the ketones to amines under the catalysis of RTA, alanine was used as amine donor, and pyruvate being the co-product. Maintaining alanine with a low concentration facilitates subsequent purification of the product. In addition, recycling of pyruvate back to alanine can reduce the inhibitory effect of by-product. Alanine with a small concentration (5 mM) was used, which is tenth of that of 2-octanone (50 mM). The conversion was catalyzed under the three-enzyme systems RTA&AlaDH + AlaDH&FDH, RTA#AlaDH + AlaDH#FDH, and RTA + AlaDH + FDH. Figure 10 shows the 2-octanone conversion versus reaction time. RTA + AlaDH + FDH (control) achieved a conversion of 32% after 2 h reaction, and 79.2% of 2-octanone was converted by RTA#AlaDH + AlaDH#FDH. In contrast, RTA&AlaDH + AlaDH&FDH achieved a 2-octanone conversion of 98.8% after 2 h reaction. For the conversion of acetophenone (Supplementary Figure S10), after 6 h reaction, 65, 45, and 18% were achieved by RTA&AlaDH + AlaDH&FDH, RTA#AlaDH + AlaDH#FDH, and RTA + AlaDH + FDH, respectively. RTA&AlaDH + AlaDH&FDH exhibited a higher catalysis efficiency than RTA#AlaDH + AlaDH#FDH, and much higher efficiency than RTA + AlaDH + FDH.

Supplementary Table S4 lists the published results using ω-transaminases combining AlaDH and FDH for the conversion of 2-octanone (50 mM). In the catalysis, alanine was used as amine donor. The amounts of alanine used are 250 mM by Mutti et al. (2011, 2012) and 280 mM by Koszelewski et al. (2010). In contrast, this work used alanine with a small concentration (5 mM), which is tenth of that of 2-octanone (50 mM). The conversion of 2-octanone achieved by this work is higher than the conversions by the three-enzyme systems of the published articles (Supplementary Table S4). Through the hetrosubunit splicing, the hetrosubunits are paired. This paves a short pathway for alanine recycling. The amount of alanine used is much less than that published, demonstrating that the transferring and recycling of alanine are more efficient than the three-enzyme systems (Supplementary Table S4). In addition, the reaction time of this work is 2 h in comparison to 24 h required by the catalysis in the published articles.

For the mixed enzymes RTA + AlaDH + FDH, the interactions between the enzymes are not specific interactions. Under shaking condition during the reaction, occasionally pairing of RTA with AlaDH and AlaDH with FDH occurred. However, the instantaneous pairing of the enzymes cannot be controlled and maintained for the whole reaction time, attributed to the non-specific interaction between the enzymes. This results in that, the transfers of alanine and pyruvate between RTA and AlaDH and NADH and NAD^+^ between AlaDH and FDH cannot be directional. In contrast, RTA&AlaDH + AlaDH&FDH and RTA#AlaDH + AlaDH#FDH achieved the paired hetrosubunits, RTA^S^-AlaDH^S^ and AlaDH^S^-FDH^S^, as illustrated in Figure 2. Based on the software (Discovery Studio), the linker length is 3.3 nm. Hence the largest distance between the pairing hetrosubunits is 6.6 nm. Thus, the hetrosubunit pairing paves short transferring-recycling pathways for transferring pyruvate and NAD^+^ and recycling alanine and NADH. Thus the transferring and recycling are directional and oriented. The hetrosubunit pairing through peptide bonds makes the splicing products become stable in comparison to the mixed enzymes. In addition, the splicing products have exhibited much higher affinity toward the model substrate 2-octanone. All these contribute to the splicing products exhibiting much higher catalysis efficiency than the mixed enzymes.

Based on the BN-PAGE analysis for RTA&AlaDH, RTA#AlaDH, AlaDH&FDH, and AlaDH#FDH, the linking mode corresponding to each band has been determined, and the fraction of each band has also been obtained. Based on the information, the number of the pairing hetersubunits RTA^S^-AlaDH^S^ in RTA&AlaDH is 28.5% higher than that in RTA#AlaDH, and the number of AlaDH^S^-FDH^S^ in AlaDH&FDH is comparable to that in AlaDH#FDH. Possibly, this is the reason that RTA&AlaDH contains more alpha-helical structure than RTA#AlaDH. It is implied that, RTA&AlaDH has more alanine→pyruvate→alanine cycles than RTA#AlaDH. Due to having more pairing hetrosubunits, RTA&AlaDH + AlaDH&FDH is more stable than RTA#AlaDH + AlaDH#FDH. In addition, RTA&AlaDH + AlaDH&FDH exhibited much higher affinity toward the substrate 2-octanone than RTA#AlaDH + Ala DH#FDH. As a result, RTA&AlaDH + AlaDH&FDH exhibited a higher catalysis efficiency than RTA#AlaDH + AlaDH#FDH.

## Conclusion

The heterodimerization of leucine zipper motifs promoted the *in vivo* hetrosubunit splicing, and the pairing enzymes RTA&AlaDH and AlaDH&FDH have been constructed. The hetrosubunit splicing has little effect on the intro-hydrogen bonding interactions, and can confer RTA&AlaDH having more alpha-helical structure and a higher stability. Ascribing to the hetrosubunit pairing through peptide bonds, the transferring pyruvate and NAD^+^ and recycling alanine and NADH are directional and oriented. In contrast to the mixed three enzymes RTA+AlaDH+FDH, the ligated enzymes RTA&AlaDH+AlaDH&FDH exhibited a much larger substrate affinity, higher stability, and significantly enhanced activity.

## Data Availability Statement

All datasets generated for this study are included in the article/Supplementary Material.

## Author Contributions

RL and WF designed the experiment study and prepared the manuscript. RL contributed the expression plasmids Z_E_-Int^C^-link-AlaDH/RTA-link-Int^N^-Z_R_-pETDuet and Z_E_-Int^C^-link-AlaDH/FDH-link-Int^N^-Z_R_-pETDuet, performed the experimental setup and procedure. RL, YC, and KD carried out the analysis and evaluation of the results. All the authors approved the final manuscript.

## Conflict of Interest

The authors declare that the research was conducted in the absence of any commercial or financial relationships that could be construed as a potential conflict of interest.
