# Supplementary material for: Peptide Bond Formation Between the Hetrosubunits of ω-Transaminase, Alanine Dehydrogenase, and Formate Dehydrogenase Through Subunit Splicing Promoted by Heterodimerization of Leucine Zipper Motifs
Source: Front Bioeng Biotechnol. 2020 Jun 30;8:686. doi: 10.3389/fbioe.2020.00686 (PMC7338344; doi:10.3389/fbioe.2020.00686)
Supplement: Supplementary file 1 [file Data_Sheet_1.docx]

Supporting Information

Peptide Bond Formation between the Hetrosubunits of ω-Transaminase, Alanine Dehydrogenase, and Formate Dehydrogenase through Subunit Splicing Promoted by Heterodimerization of Leucine Zipper Motifs

Rong Li, Yao Chen, Ku Du, Wei Feng

Department of Biological Engineering, Beijing University of Chemical Technology, Beijing 100029, China.

**Blue native PAGE.** Blue Native PAGE (BN-PAGE) was performed at room temperature utilizing 3.5% stacking gel and 4–11% separating gel with the 0.15×10×10 cm gel dimensions. The gel compositions and the cathode and anode buffers were prepared prior to use. The marker protein standard (Invitrogen) was comprised by seven protein molecular weights from 66~1236 kDa. 30 ng enzyme sample was added to each lane with an enzyme concentration of 0.5 mg/mL. The BN-PAGE electrophoresis was performed at 100 V from the beginning. After enzyme reaching the separating gel, the voltage was changed to 200 V and run until the enzyme front reached the end of the gel.

The obtained gels were photoed and high-resolution images (600 dpi) were obtained. The Gel-Pro Analyzer software program was used to estimate optical densitometry for determining relative abundances of the splicing product fractions. By comparing the electrophoretic mobility of the splicing product fractions with that of Mark proteins, the apparent molecular weights of the splicing product fractions were determined. The relative mobility was computed by measuring the distance from the top of the gel to the middle of the dye front. The relative mobility versus molecular weight for the Mark proteins is plotted as shown in Figures S1 and S3.

**Circular dichroism (CD) spectra.** By measuring CD spectra on a JASCO J-810 CD instrument, the change of secondary structures of the enzymes was monitored. The scanning was performed at 25 °C with a scan rate of 100 nm/min with a bandwidth of 1.0 nm and cell length of 1 cm. The concentration of the enzymes was 0.2 mg/mL. The samples were prepared by dissolving the enzymes in PBS which was measured as a control. Averaged spectra were obtained via repeating five times scan.

**Fluorescence measurements.** Fluorescence spectra was measured on a spectrophotometer (F-7000) with usage of 1 cm pathlength cuvette. Emission spectra of the tryptophan was recorded for wavelength ranging from 290 to 400 nm, using an excitation wavelength of 295 nm. In addition, the slits for excitation and emission were decided at 5 nm. The scan speed was 1200 nm/min. For all the samples, the background fluorescence was subscribed to correct the fluorescence spectra. Buffer A (pH 7.5) contained 50 mM potassium phosphate and 2 mM EDTA. Buffer B (pH 7.5) consisted of 50 mM potassium phosphate, 2 mM EDTA, and 2 M urea. The enzyme solutions were diluted by buffer A and buffer B to prepare samples, incubated at 4 ^0^C overnight. Acrylamide of various concentration was used for fluorescence quenching.

**Table S1**. Primers for construction of the plasmids

| Primers | Sequence (5’-3’) |
| --- | --- |
| RTA-fp | GGAATTCCATATGGTTGCGTTCTCTGCGGACACC |
| RTA-rp | CGGGGTACCGTACTGAACCGGGGTCAGCAG |
| AlaDH-fp | CGCGGATCCATGATCATAGGGGTTCCTAAAG |
| AlaDH-rp | CCGGAATTCTCGTGGGCGGTGTCT |
| FDH-fp | GGAATTCCATATGATGGCAAAGGTCCT |
| FDH-rp | CGGGGTACCGGCGGCCTCTTCCG |
| Z_E_-Int^C^-fp | CATGCCATGGATGCTGGAGATCGAAG |
| Z_E_-Int^C^-rp | CGCGGATCCCGGTGGCGGCGGTTC |
| Int^N^-Z_R_-fp | GGGGTACCGAGCCACCACCGCCG |
| Int^N^-Z_R_-rp | CCGCTCGAGTAAAGGTCCATAGCG |
| Int^C^-fp | CATGCCATGGGCATGATCAAAATAG |
| Int^C^-rp | CGCGGATCCCGGTGGCGGCGG |
| Int^N^-fp | CGGGGTACCGAGCCACCACCGCC |
| Int^N^-rp | CCGCTCGAGATTCGGCAAATTA |

Amplification conditions

| Plasmid DNA | 100 ng |
| --- | --- |
| Primer AlaDH-fp | 20 μL |
| Primer AlaDH-rp | 20 μL |
| 10 mM dNTP | 20 μL |
| KOD DNA polymerase | 5 μL |
| 5 × KOD Buffer | 40 μL |
| Double distilled H2O | 85 μL |
| Cycling parameters |  |
| 94 ^0^C | 4 min |
| 94 ^0^C | 42 s |
| 58 ^0^C | 42 s |
| 72 ^0^C | 2 min |
| Repeating the above 2 **~** 4 steps for 29 times | |
| 72 ^0^C | 10 min |

**Table S2**. Ligation modes for the splicing products

| Splicing products | Band | Apparent molecular weight (kDa) | Theoretic molecular weight (kDa) | Ligation mode |
| --- | --- | --- | --- | --- |
| RTA&AlaDH | Ⅰ | 254.5 | 231.2 | RTA~AlaDH |
|  | Ⅱ | 311.9 | 303.6 | RTA~AlaDH~RTA |
|  | Ⅲ | 388.0 | 390 | AlaDH~RTA~AlaDH |
|  | Ⅳ | 422.4 | 462.4 | (RTA~AlaDH)_2_ |
|  | Ⅴ | 480.7 | 534.8 | (RTA~AlaDH)_2_~RTA |
|  | Ⅵ | 564.9 | 621.2 | AlaDH~(RTA~AlaDH)_2_ |
|  | Ⅶ | 674.2 | 693.6 | (RTA~AlaDH)_3_ |
|  | VIII | 741.3 | 766 | (RTA~AlaDH)_3_~RTA |
| RTA#AlaDH | Ⅰ | 271.2 | 231.2 | RTA~AlaDH |
|  | Ⅱ | 346.7 | 390 | AlaDH~RTA~AlaDH |
|  | Ⅲ | 435.1 | 462.4 | (RTA~AlaDH)_2_ |
|  | Ⅳ | 761.7 | 766 | (RTA~AlaDH)_3_~RTA |
| AlaDH&FDH | Ⅰ | 210.6 | 166.2 | FDH~AlaDH |
|  | Ⅱ | 278.7 | 253 | FDH~AlaDH~FDH |
|  | Ⅲ | 327.0 | 332.4 | (FDH~AlaDH)_2_ |
|  | Ⅳ | 399.4 | 411.8 | AlaDH~(FDH~AlaDH)_2_ |
|  | Ⅴ | 469.7 | 498.6 | (FDH~AlaDH)_3_ |
|  | Ⅵ | 533.8 | 578 | AlaDH~(FDH~AlaDH)_3_ |
|  | Ⅶ | 665.1 | 664.8 | (FDH~AlaDH)_4_ |
| AlaDH#FDH | Ⅰ | 205.8 | 166.2 | FDH~AlaDH |
|  | Ⅱ | 280.9 | 253.0 | FDH~AlaDH~FDH |
|  | Ⅲ | 331.6 | 332.4 | (FDH~AlaDH)_2_ |
|  | Ⅳ | 403.8 | 411.8 | AlaDH~(FDH~AlaDH)_2_ |
|  | Ⅴ | 679.3 | 664.8 | (FDH~AlaDH)_4_ |

**Table S3.** Assignment of the cross-peaks found on the asynchronous correlation maps of Figure 7.

| Enzymes | Faster exchanging  component (cm^-1^) | Slower exchanging  component (cm^-1^) |
| --- | --- | --- |
| RTA&AlaDH | 1450 (u) | 1650 (α) |
|  | 1450 (u) | 1610 (β) |
| RTA#AlaDH | 1430 (u) | 1639 (α) |
|  | 1437 (u) | 1665 (β) |
| RTA+AlaDH | 1425 (u) | 1645 (α) |
|  | 1427 (u) | 1680 (β) |

α: α−helix; β: β−sheet; u: unordered structure.

Table S4. Conversion of 2-Octanone under catalysis of various enzyme systems

| Enzyme systems | Substrate  (mM) | Alanine  (mM) | Reaction time (h) | Conversion  (%) | | Ref |
| --- | --- | --- | --- | --- | --- | --- |
| BM-ωTA+  AlaDH+FDH | 50 | 250 | 24 | | 64 | [a] |
| AD-ωTA+  AlaDH+FDH | 50 | 250 | 24 | | 5 | [a] |
| ArS-ωTA+  AlaDH+FDH | 50 | 250 | 24 | | 4 | [a] |
| CV-ωTA+  AlaDH+FDH | 50 | 250 | 24 | | 33 | [a] |
| ArR-ωTA+  AlaDH+FDH | 50 | 250 | 24 | | 75 | [b] |
| AT-ωTA+  AlaDH+FDH | 50 | 250 | 24 | | 92 | [b] |
| HN-ωTA+  AlaDH+FDH | 50 | 250 | 24 | | 65 | [b] |
| His-Vf-ωTA+  AlaDH+FDH | 50 | 250 | 24 | | 80 | [c] |
| Strep-PD-ωTA+AlaDH+FDH | 50 | 250 | 24 | | 42 | [c] |
| PF-ωTA+  AlaDH+FDH | 50 | 250 | 24 | | 78 | [c] |
| RTA&AlaDH+  AlaDH&FDH | 50 | 5 | 2 | | 98.8 | This work |
| RTA#AlaDH+  AlaDH#FDH | 50 | 5 | 2 | | 79.2 | This work |

[a] Koszelewski, D., Gçritzer, M., Clay, D., Seisser, B., and Kroutil, W. (2010). Synthesis of optically active amines employing recombinant ω-transaminases in E. coli cells. *ChemCatChem*. 2, 73-77. doi: 10.1002/cctc.200900220

[b] Mutti, G. F., Fuchs, S. C., Pressnitz, D., Sattler, H. J., and Kroutil, W. (2011). Stereoselectivity of four (R)-selective transaminases for the asymmetric amination of ketones. *Adv. Synth. Catal.* 353, 3227-3233. doi: 10.1002/adsc.201100558

[c] Mutti, G. F., Fuchs, S. C., Pressnitz, D., Turrini, G. N., Sattler, H. J., Lerchner. A., et al. (2012). Amination of ketones by employing two new (S)-selective ω-transaminases and the His-tagged ω-TA from vibrio fluvialis. *Eur. J. Org. Chem.* 1003-1007. doi: 10.1002/ejoc.201101476


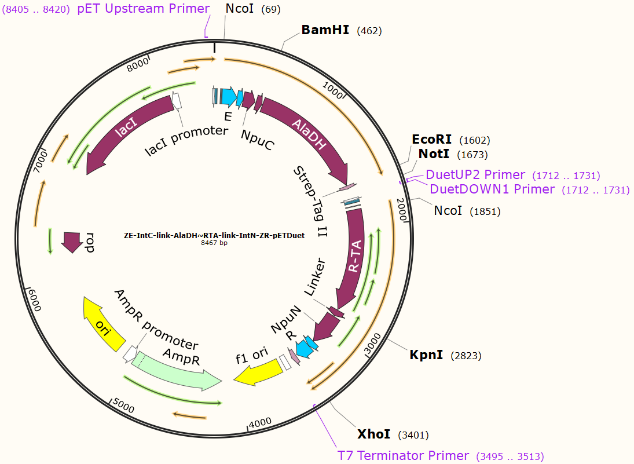

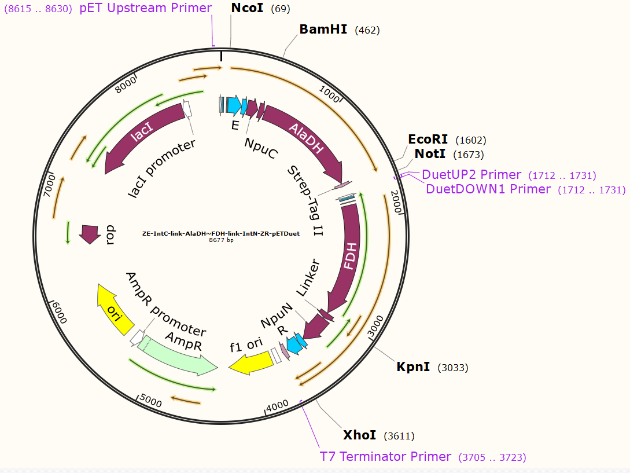


a b


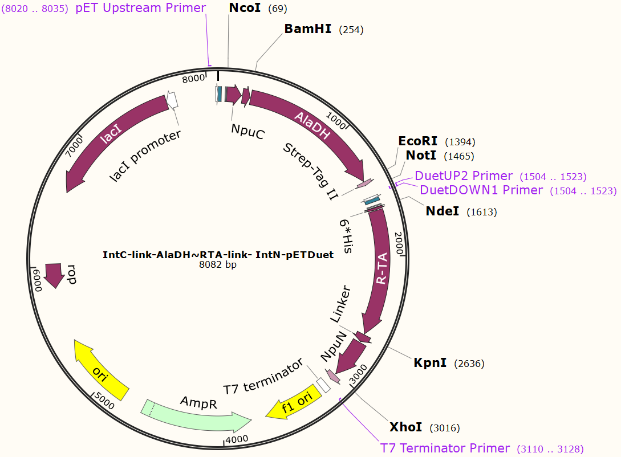

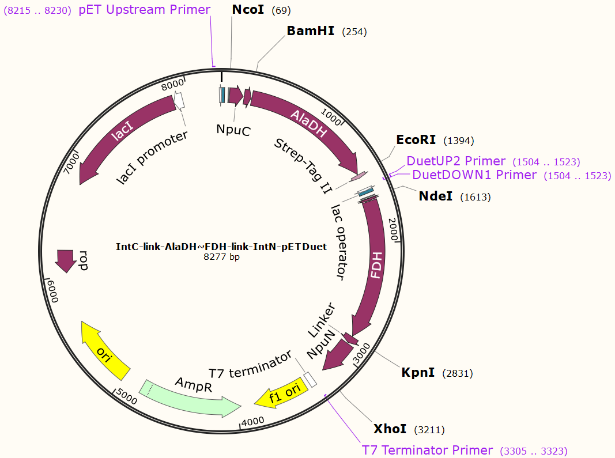


c d

**Figure S1**. Plasmids for the in vivo subunit splicing.

(a) Z_E_-Int^C^-link-AlaDH/RTA(FDH)-link-Int^N^-Z_R_-pETDuet; (b) Z_E_-Int^C^-link-AlaDH/FDH-link-Int^N^-Z_R_-pETDuet; (c) Int^C^-link-AlaDH/RTA-link-Int^N^-pETDuet; (d) Int^C^-link-AlaDH/FDH-link-Int^N^-Z_R_-pETDuet.


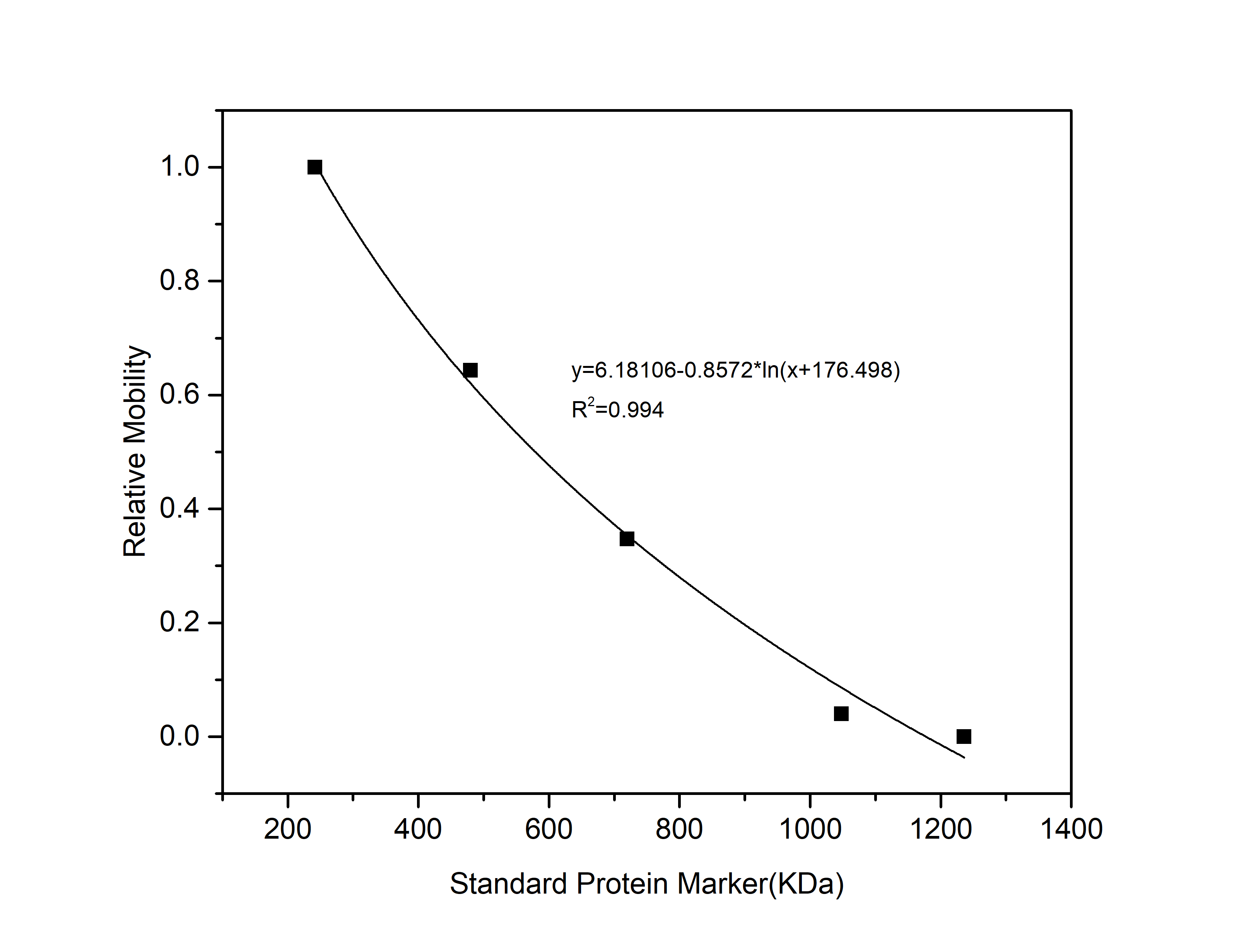


**Figure S2**. The relative mobility versus molecular weight for the Mark proteins for estimation of the apparent molecular weights of the fractions of RTA&AlaDH and RTA#AlaDH


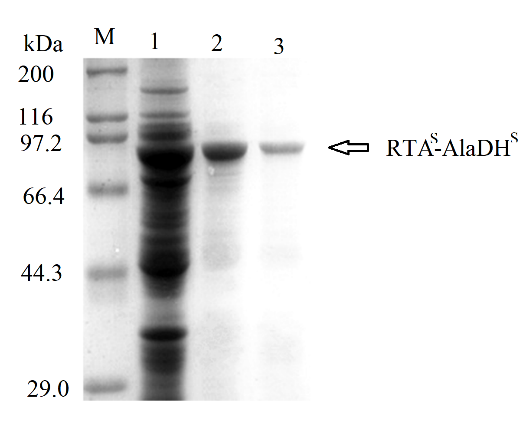

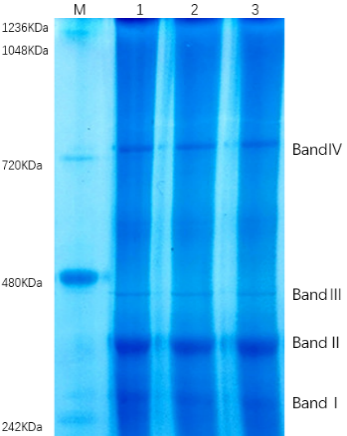


a b


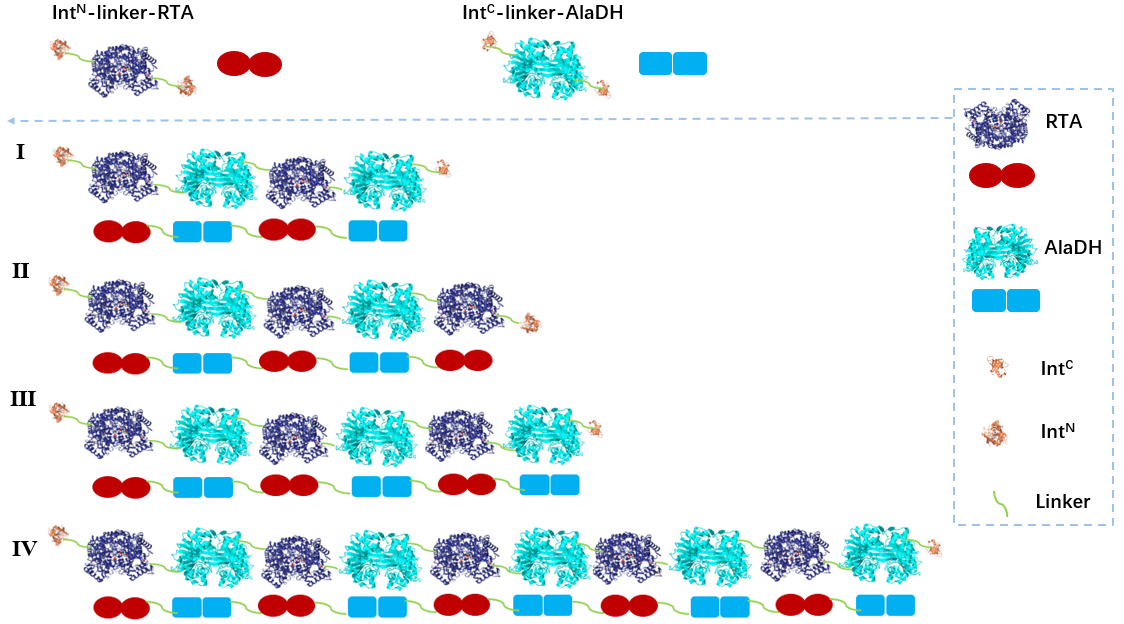


c

**Figure S3.** (a) SDS-PAGE for RTA#AlaDH. Lane 3 for RTA#AlaDH.

(b) Analysis of the splicing product RTA#AlaDH by BN-PAGE.

Lane M: marker.

(c) Schematic diagram representing the RTA#AlaDH fractions derived from the ligation modes of RTA~AlaDH (band I), AlaDH~RTA~AlaDH (band II), RTA~AlaDH~RTA~AlaDH (band III), RTA~AlaDH~RTA~AlaDH~RTA~AlaDH~RTA (band IV).

产物

产物

产物


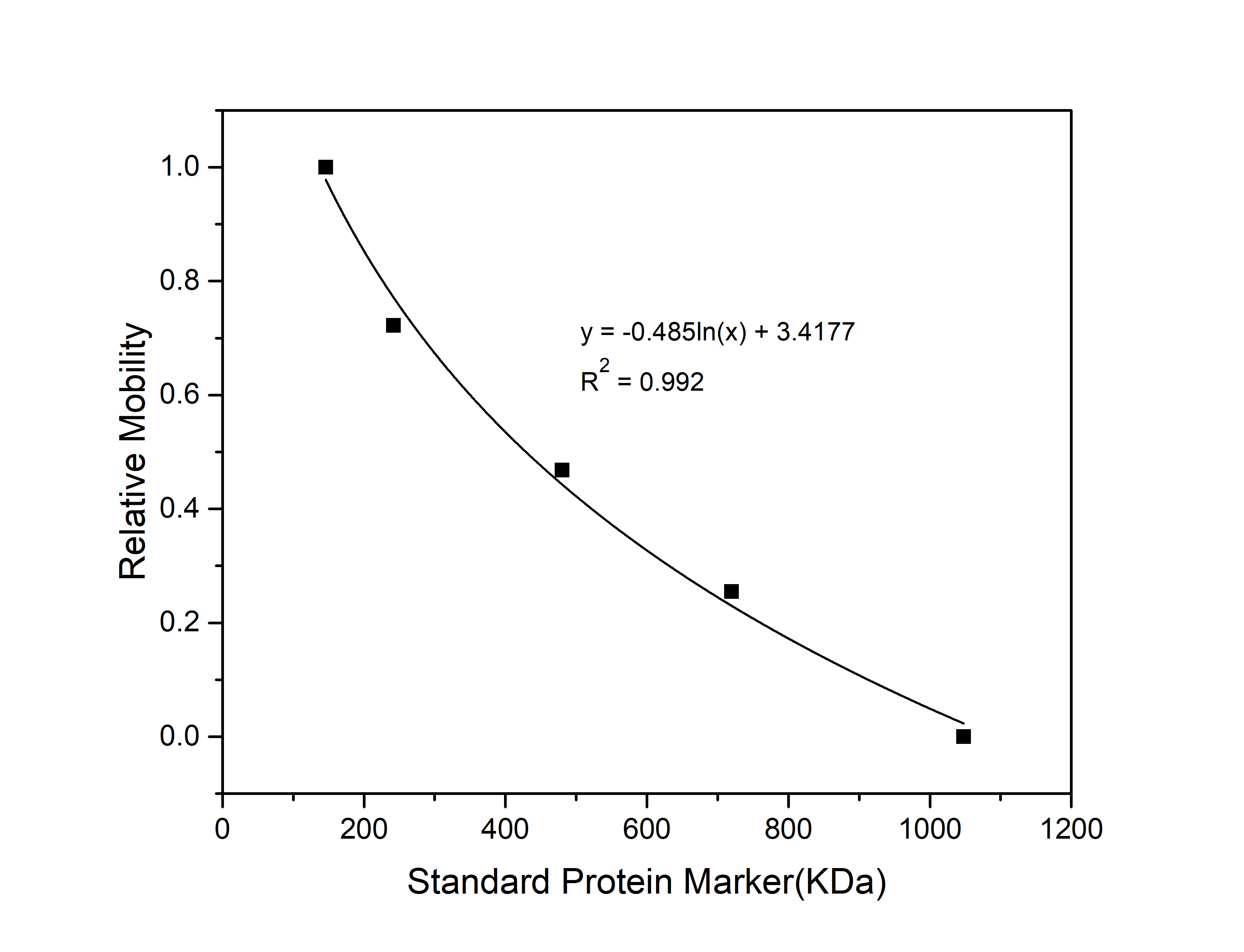


**Figure S4**. The relative mobility versus molecular weight for the Mark proteins for estimation of the apparent molecular weights of the fractions of AlaDH&FDH and AlaDH#FDH


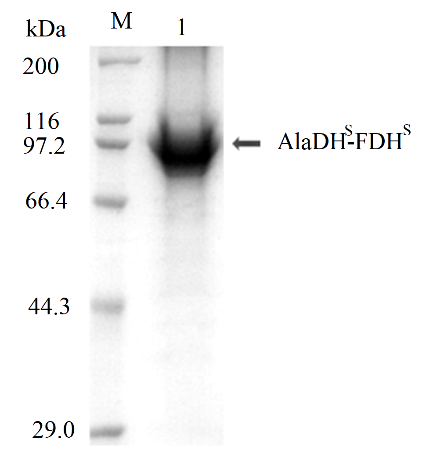

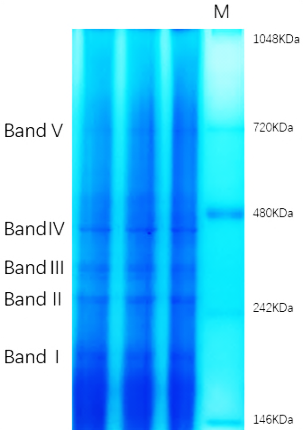


a b


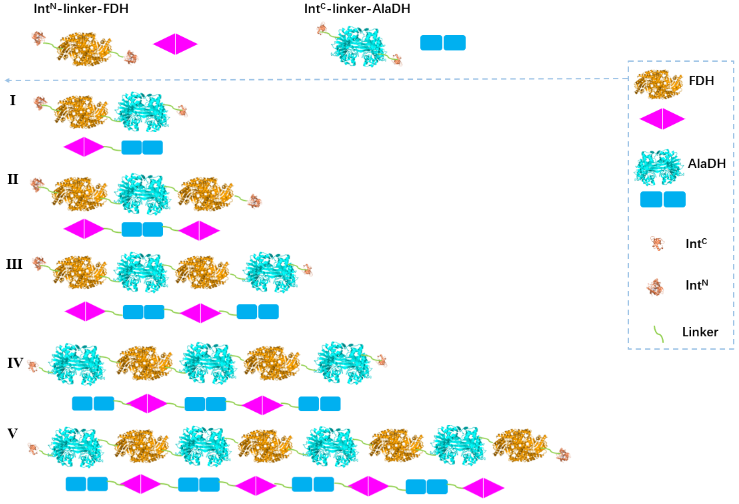


c

**Figure S5**. (a) Analysis of the splicing product AlaDH&FDH by BN-PAGE showing seven bands. Lane M: marker. Lanes 1, 2, 3 are for three samples.

(b) Schematic diagram representing the AlaDH&FDH fractions derived from the ligation modes of FDH**~**AlaDH (band I), FDH**~**AlaDH**~**FDH (band II), FDH**~**AlaDH**~**FDH**~**AlaDH (band III), AlaDH**~**FDH**~**AlaDH**~**FDH~AlaDH (band IV), AlaDH**~**FDH**~**AlaDH**~**FDH~AlaDH**~**FDH (band V), AlaDH**~**FDH**~**AlaDH**~** FDH**~**AlaDH**~**FDH**~**AlaDH (band VI), AlaDH**~**FDH**~**AlaDH**~**FDH**~**AlaDH**~**FDH **~**AlaDH**~**FDH (band VII).

产物

产物

产物


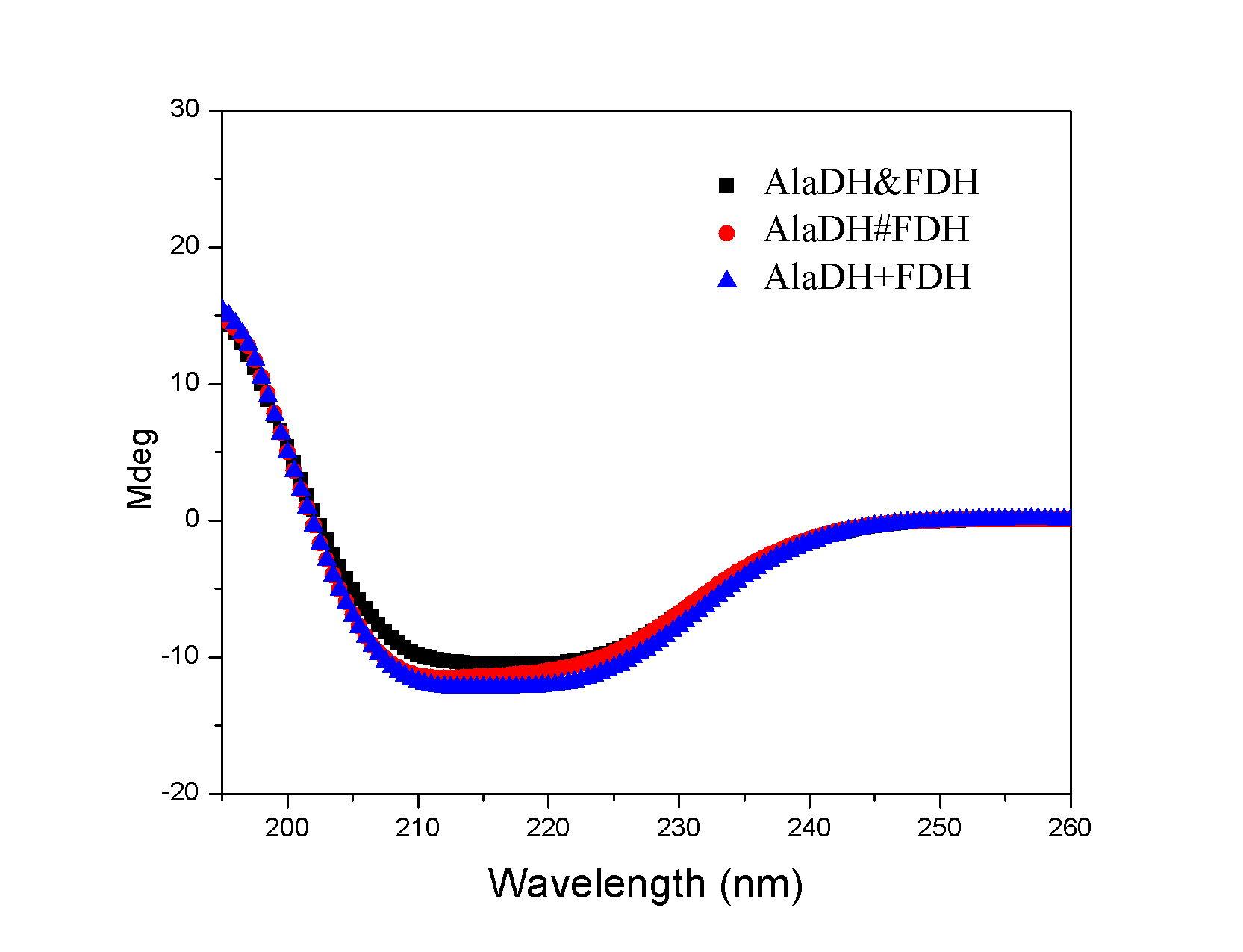


**Figure S6**. CD spectra for the splicing products AlaDH&FDH and AlaDH**#**FDH and the mixed enzymes AlaDH**+**FDH


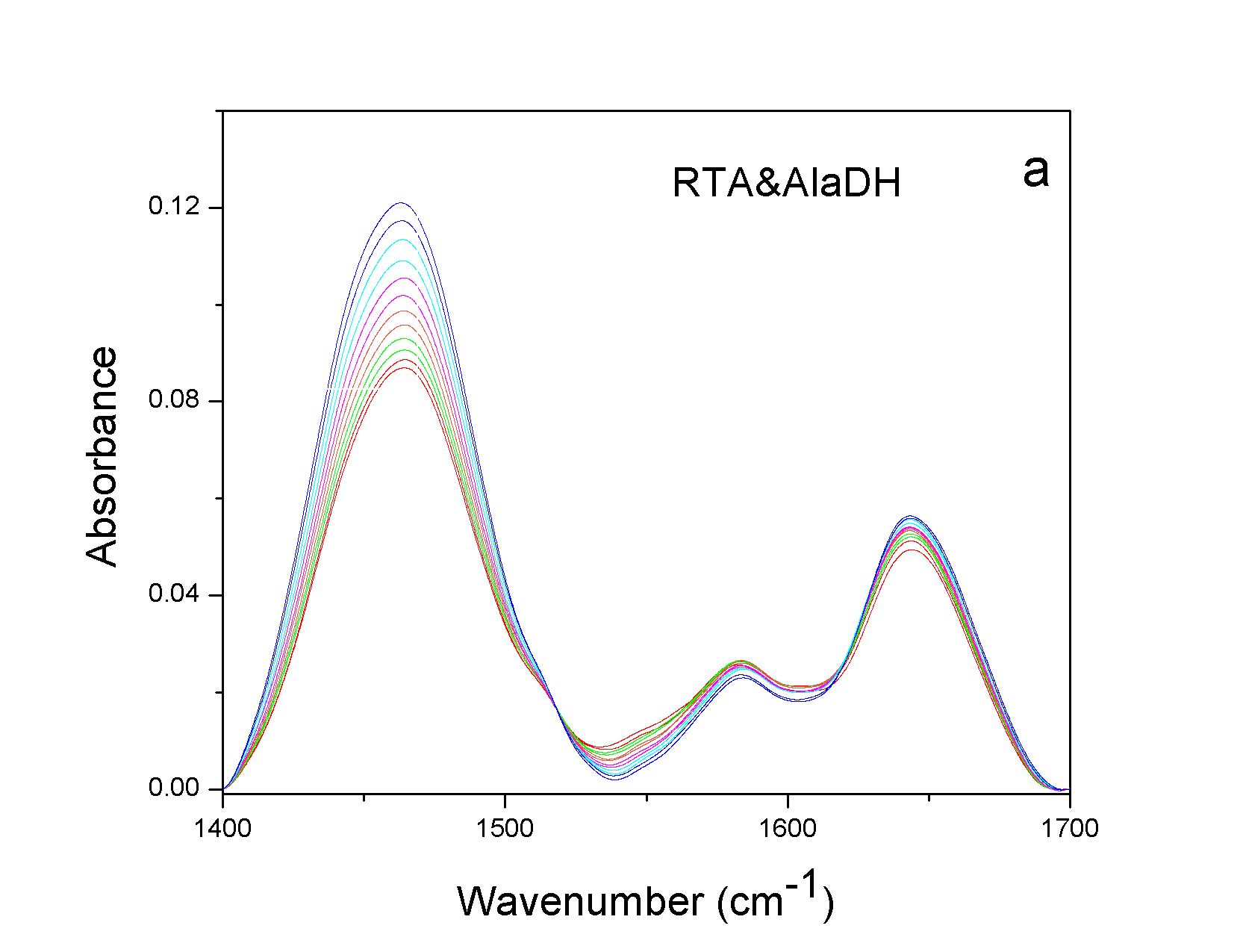

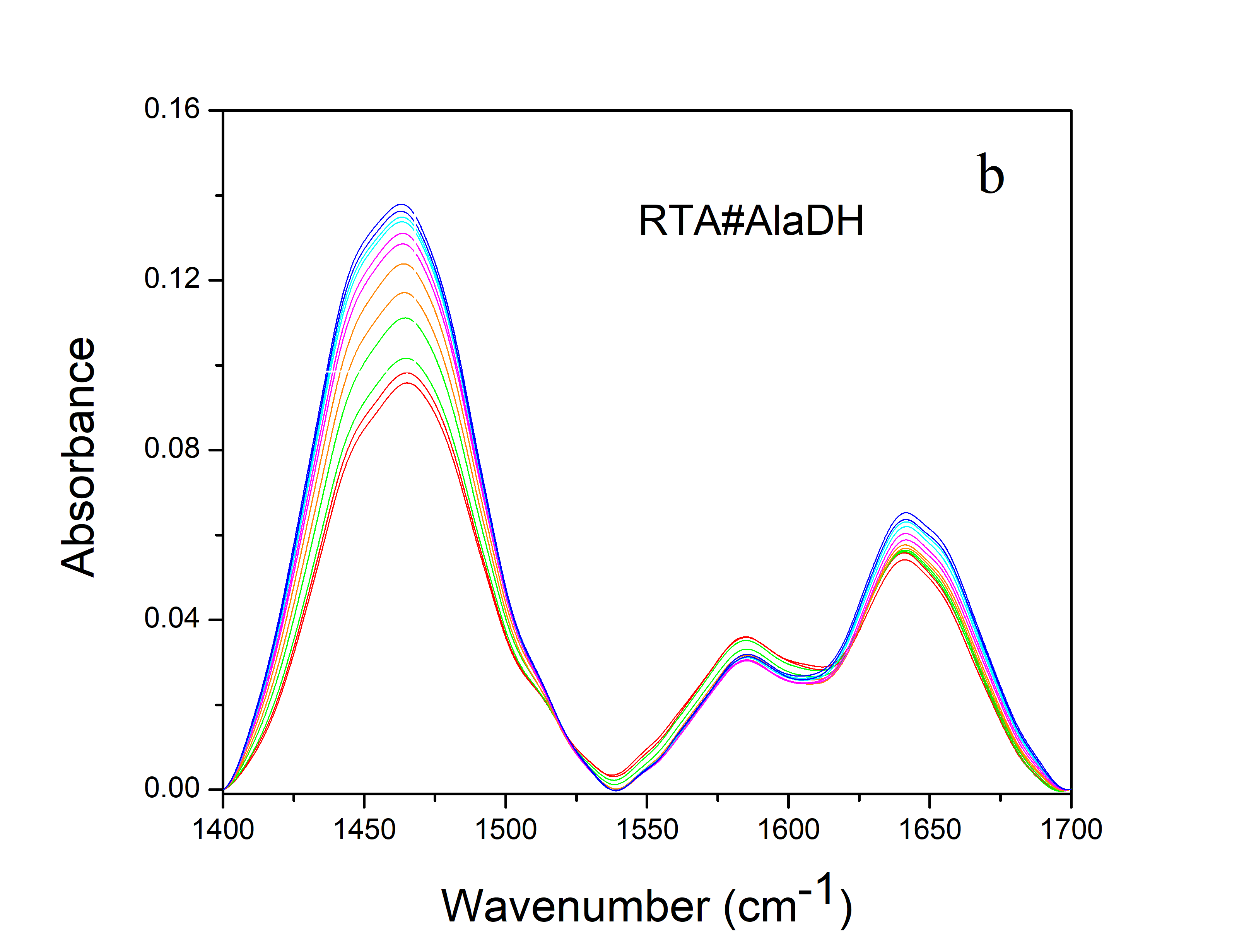

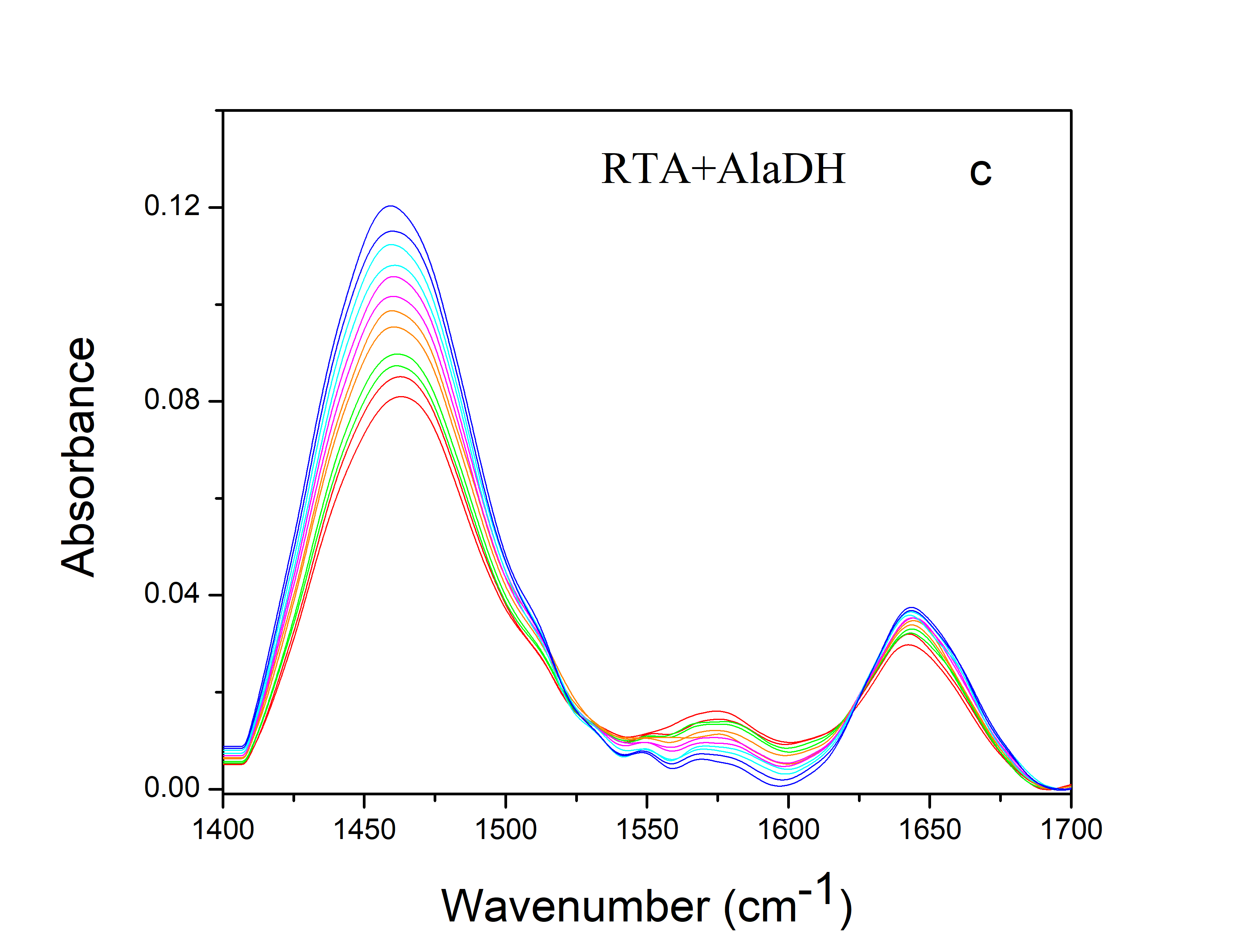


**Figure S7**. Infrared spectra as a function of the deuteration time

1. RTA&AlaDH; (b) RTA#AlaDH; (c) RTA+AlaDH


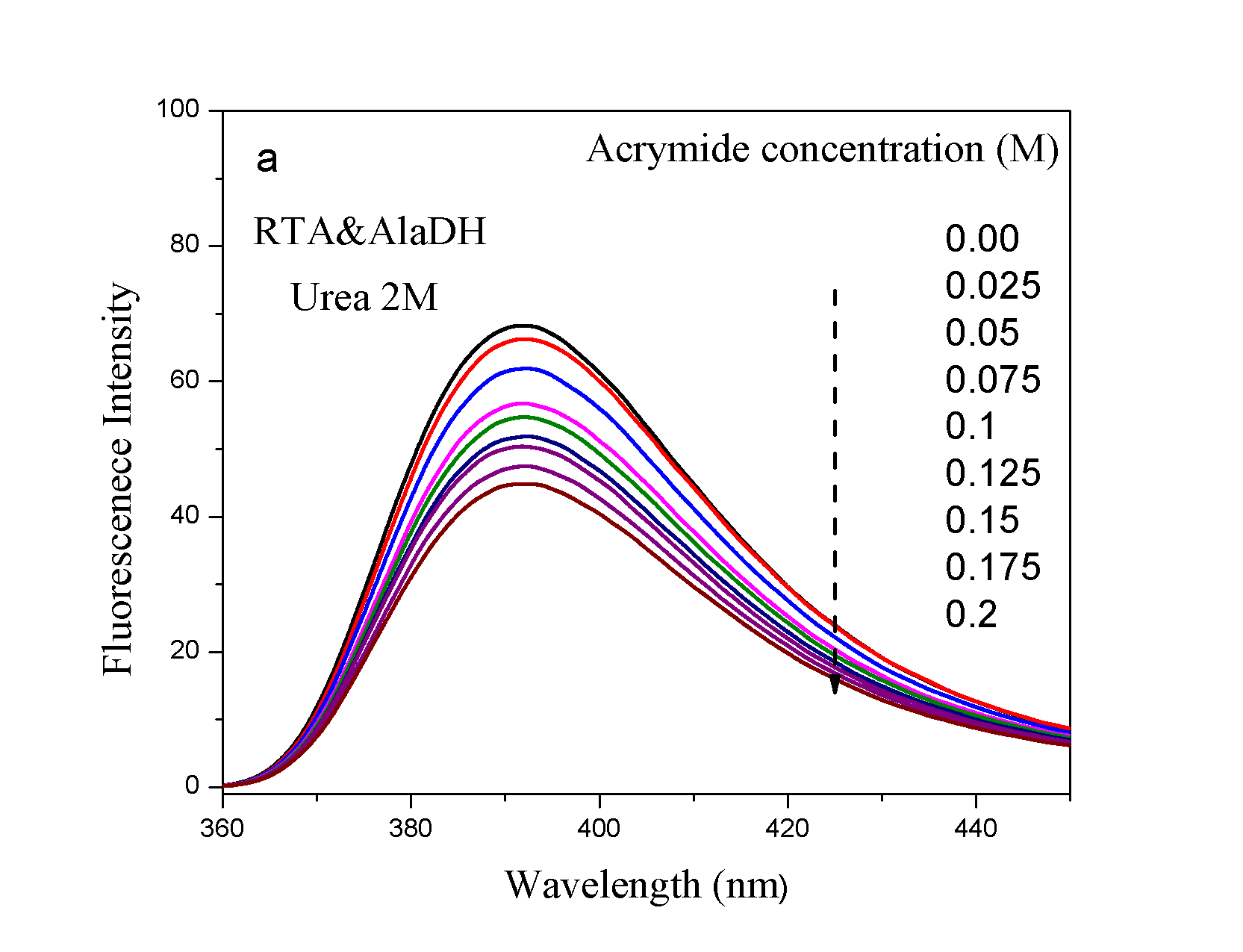

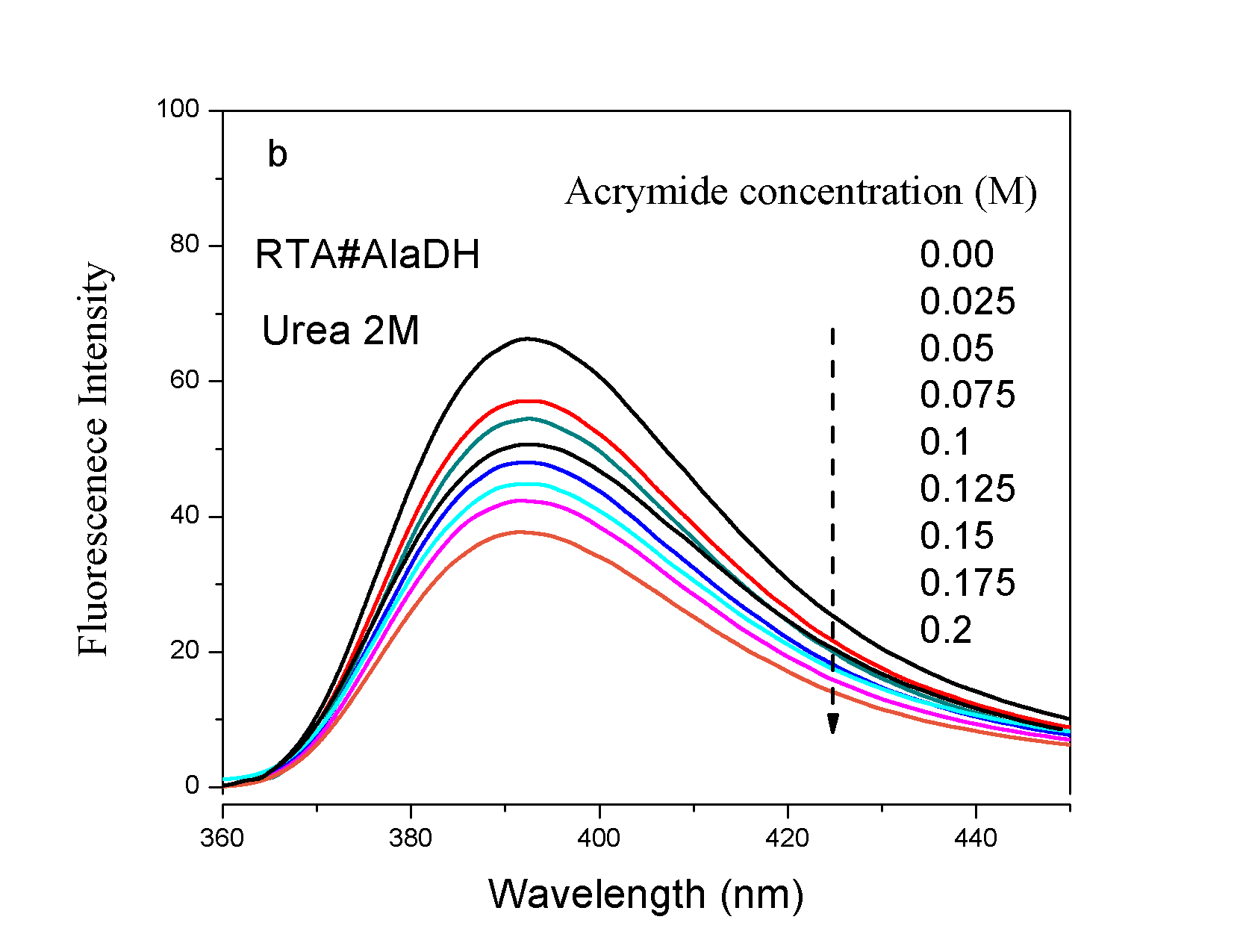

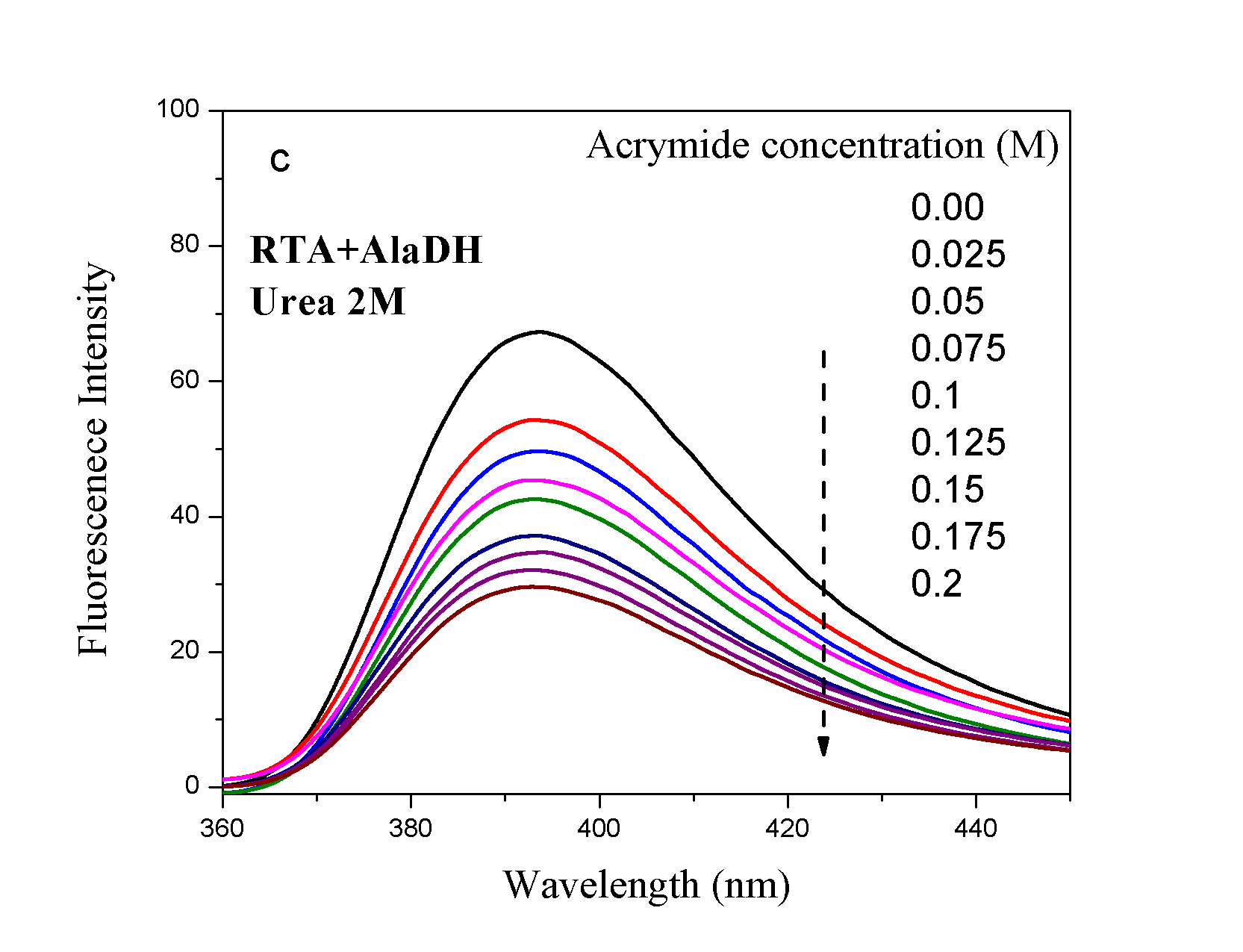


**Figure S8**. Change of fluorescence intensity of the two-enzyme systems with acrylamide concentration (M).


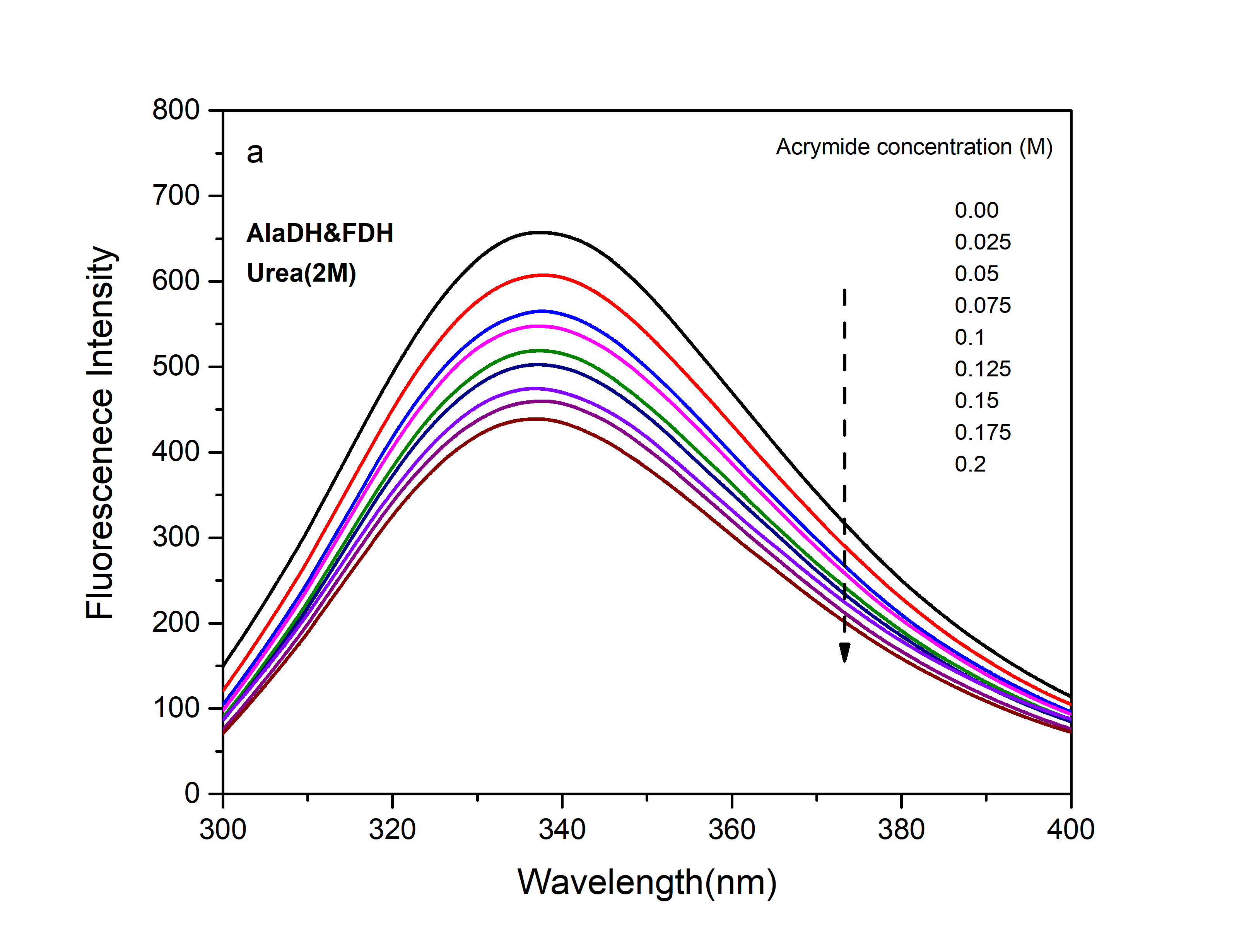

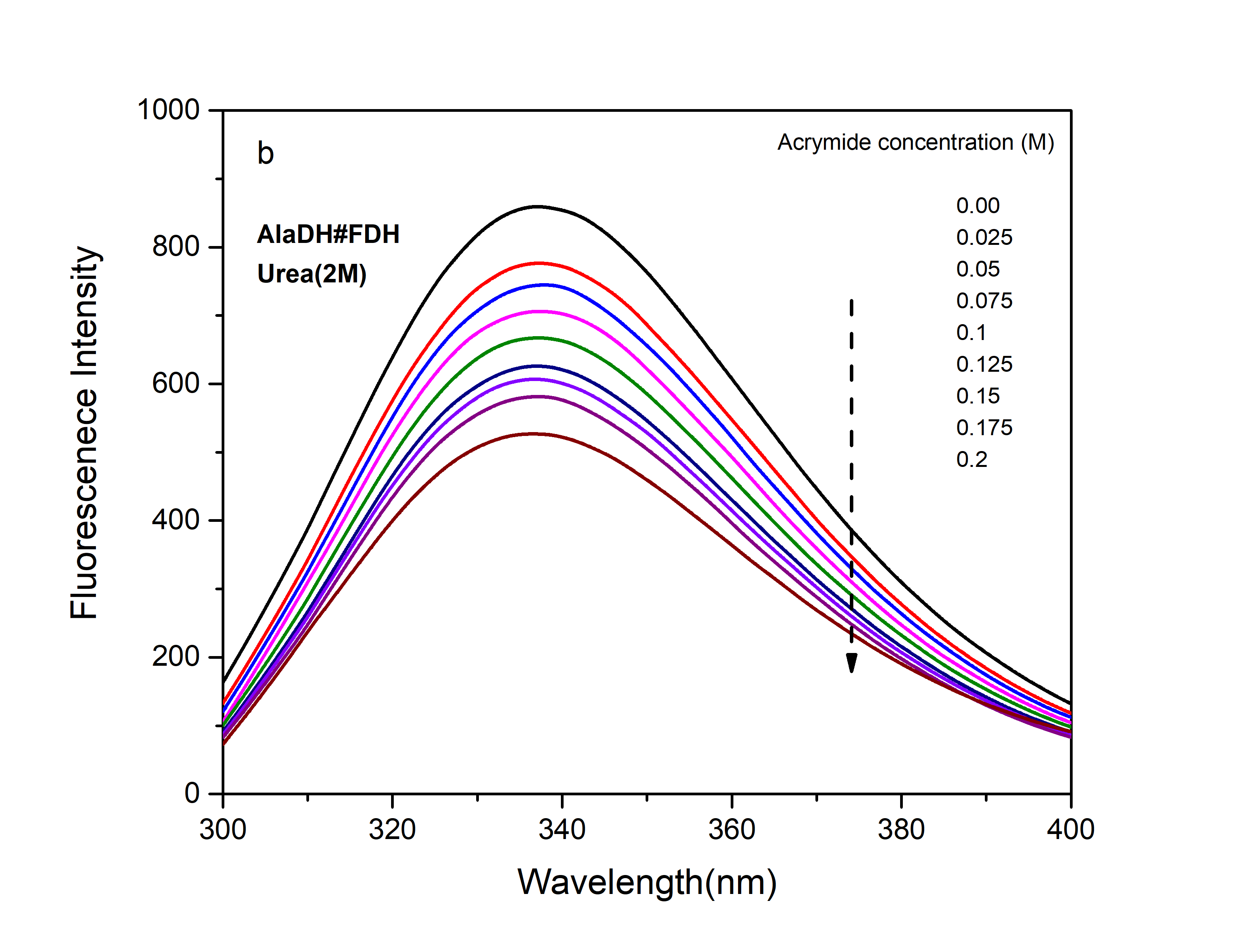


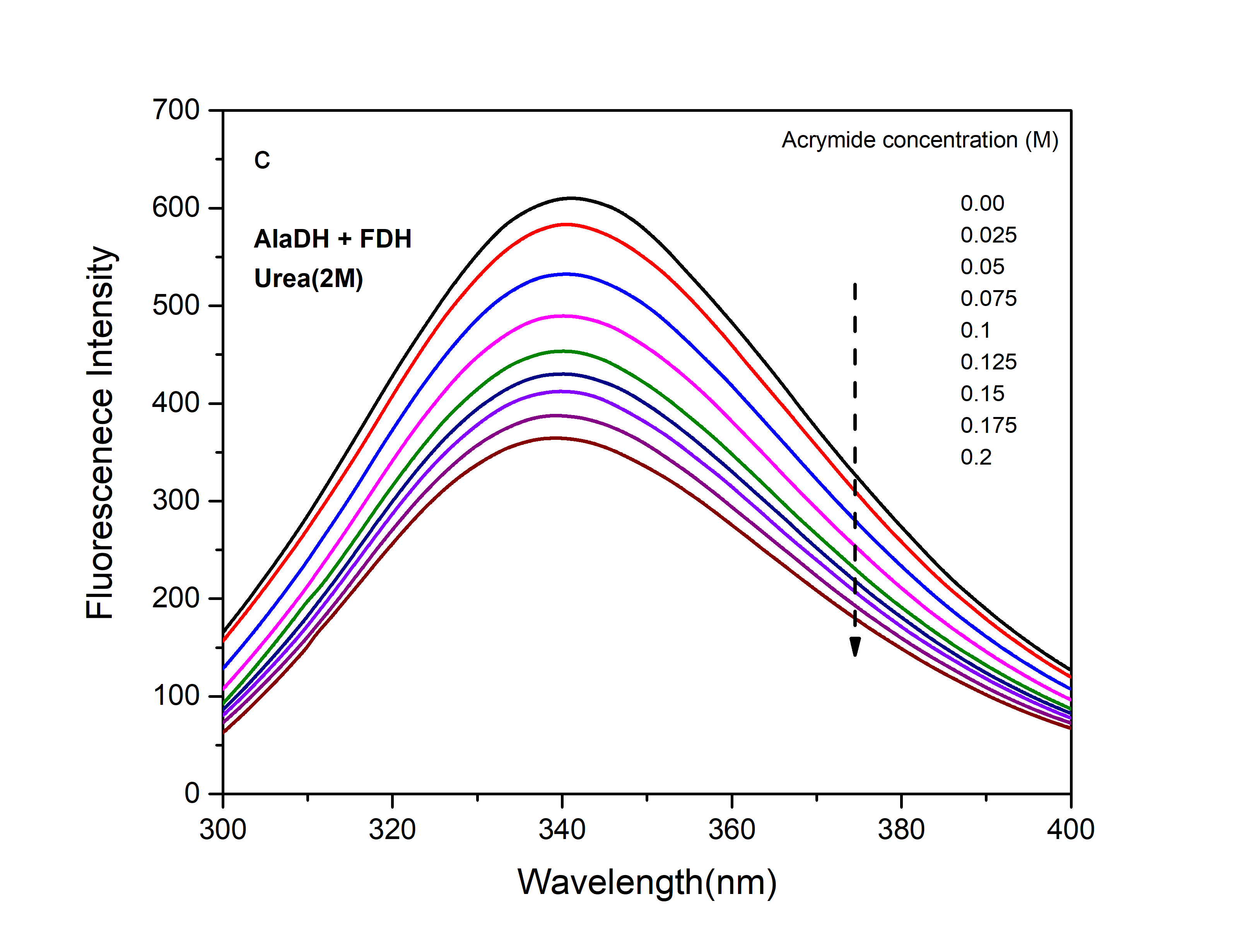

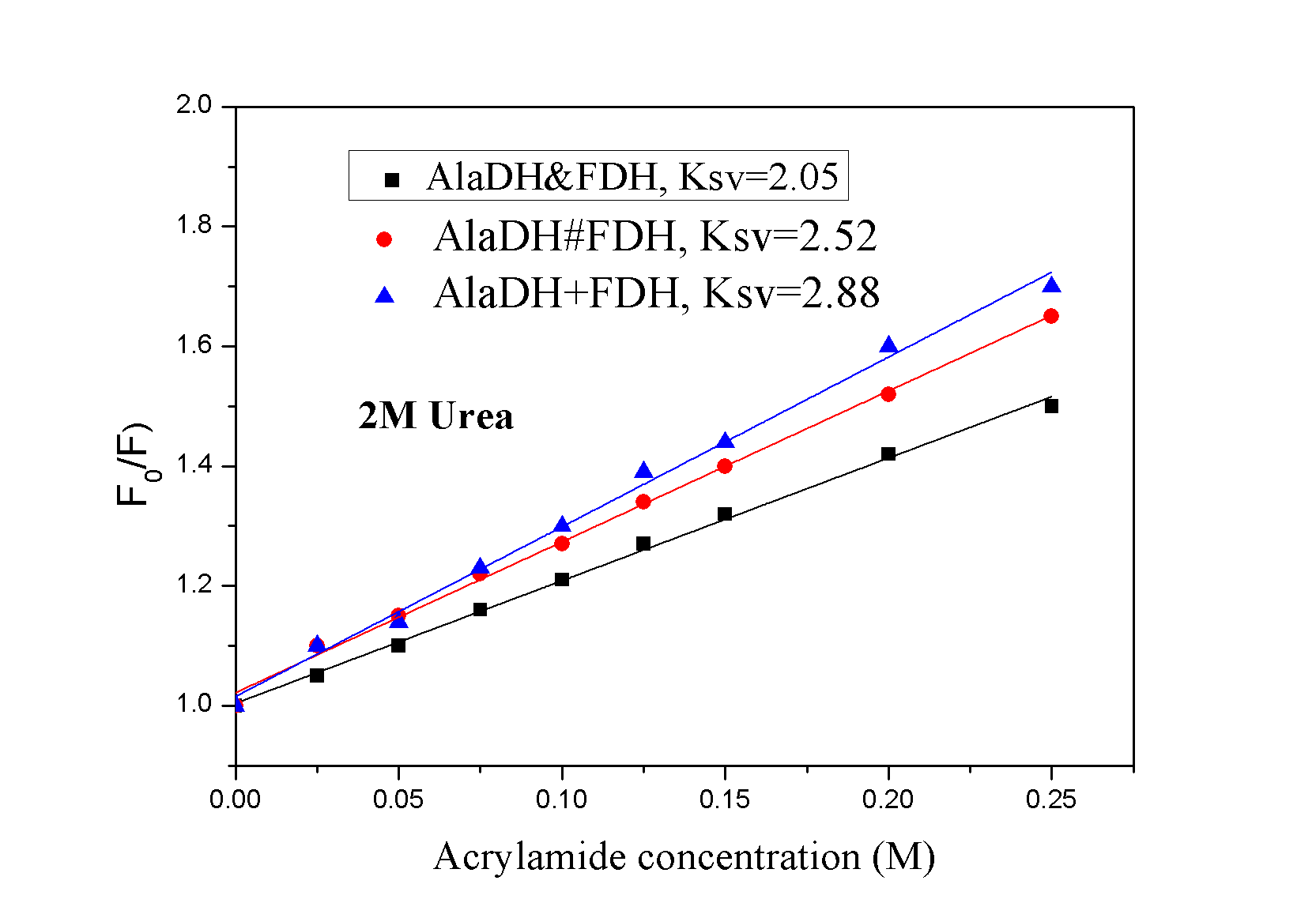


d

**Figure S9**. (a, b, c) Change of fluorescence intensity of the two-enzyme systems with acrylamide concentration (M). (d) The *K_SV_* values for AlaDH&FDH, AlaDH**#**FDH, and AlaDH**+**FDH are 2.05, 2.52, and 2.88, respectively. Their stabilities are in the sequence AlaDH&FDH **>** AlaDH**#**FDH **>** AlaDH**+**FDH.


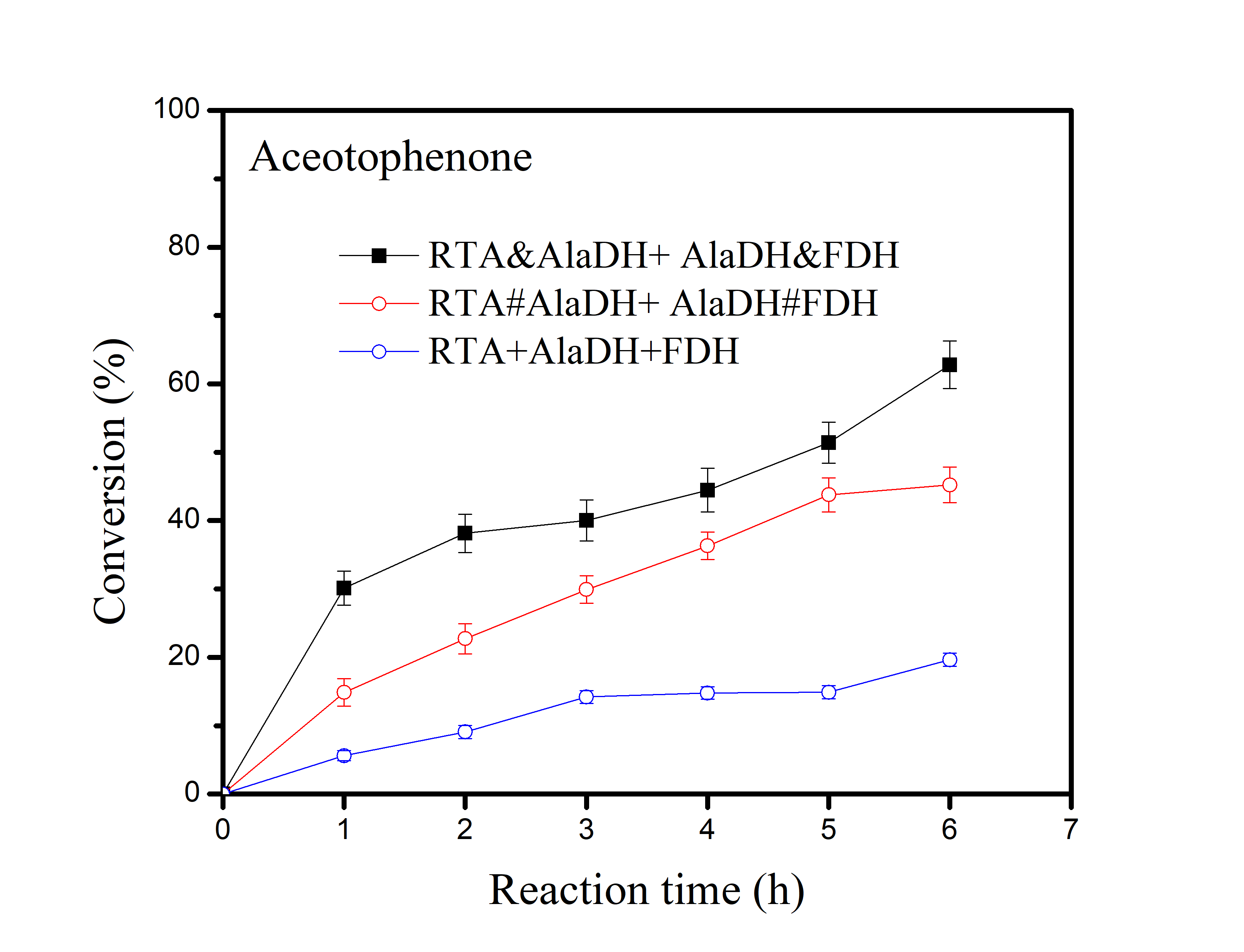


**Figure S10**. Conversion of acetophenone under the catalysis of RTA&AlaDH+AlaDH&FDH, RTA**#**AlaDH+AlaDH#FDH, and RTA**+**AlaDH+FDH.
